# Exploring the Feasibility of a 5-Week mHealth Intervention to Enhance Physical Activity and an Active, Healthy Lifestyle in Community-Dwelling Older Adults: Mixed Methods Study

**DOI:** 10.2196/63348

**Published:** 2025-01-27

**Authors:** Kim Daniels, Sharona Vonck, Jolien Robijns, Kirsten Quadflieg, Jochen Bergs, Annemie Spooren, Dominique Hansen, Bruno Bonnechère

**Affiliations:** 1 Centre of Expertise in Care Innovation, Department of PXL – Healthcare PXL University of Applied Sciences and Arts Hasselt Belgium; 2 REVAL Rehabilitation Research Center Faculty of Rehabilitation Sciences Hasselt University Diepenbeek Belgium; 3 THINK3 Simulation & Innovation Lab Faculty of Medicine and Life Sciences Hasselt University Diepenbeek Belgium; 4 BIOMED Faculty of Medicine and Life Sciences Hasselt University Diepenbeek Belgium; 5 Technology-Supported and Data-Driven Rehabilitation Data Sciences Institute Hasselt University Diepenbeek Belgium

**Keywords:** mobile health, mHealth, feasibility, physical activity, older adults, health promotion, usability, mobile phone

## Abstract

**Background:**

Advancements in mobile technology have paved the way for innovative interventions aimed at promoting physical activity (PA).

**Objective:**

The main objective of this feasibility study was to assess the feasibility, usability, and acceptability of the More In Action (MIA) app, designed to promote PA among older adults. MIA offers 7 features: personalized tips, PA literacy, guided peer workouts, a community calendar, a personal activity diary, a progression monitor, and a chatbot.

**Methods:**

Our study used a mixed methods approach to evaluate the MIA app’s acceptability, feasibility, and usability. First, a *think-aloud* method was used to provide immediate feedback during initial app use. Participants then integrated the app into their daily activities for 5 weeks. Behavioral patterns such as user session duration, feature use frequency, and navigation paths were analyzed, focusing on engagement metrics and user interactions. User satisfaction was assessed using the System Usability Scale, Net Promoter Score, and Customer Satisfaction Score. Qualitative data from focus groups conducted after the 5-week intervention helped gather insights into user experiences. Participants were recruited using a combination of web-based and offline strategies, including social media outreach, newspaper advertisements, and presentations at older adult organizations and local community services. Our target group consisted of native Dutch-speaking older adults aged >65 years who were not affected by severe illnesses. Initial assessments and focus groups were conducted in person, whereas the intervention itself was web based.

**Results:**

The study involved 30 participants with an average age of 70.3 (SD 4.8) years, of whom 57% (17/30) were female. The app received positive ratings, with a System Usability Scale score of 77.4 and a Customer Satisfaction Score of 86.6%. Analysis showed general satisfaction with the app’s workout videos, which were used in 585 sessions with a median duration of 14 (IQR 0-34) minutes per day. The Net Promoter Score was 33.34, indicating a good level of customer loyalty. Qualitative feedback highlighted the need for improvements in navigation, content relevance, and social engagement features, with suggestions for better calendar visibility, workout customization, and enhanced social features. Overall, the app demonstrated high usability and satisfaction, with near-daily engagement from participants.

**Conclusions:**

The MIA app shows significant potential for promoting PA among older adults, evidenced by its high usability and satisfaction scores. Participants engaged with the app nearly daily, particularly appreciating the workout videos and educational content. Future enhancements should focus on better calendar visibility, workout customization, and integrating social networking features to foster community and support. In addition, incorporating wearable device integration and predictive analytics could provide real-time health data, optimizing activity recommendations and health monitoring. These enhancements will ensure that the app remains user-friendly, relevant, and sustainable, promoting sustained PA and healthy behaviors among older adults.

**Trial Registration:**

ClinicalTrials.gov NCT05650515; https://clinicaltrials.gov/study/NCT05650515

## Introduction

### Background

Ensuring the well-being of the global aging population is a pressing concern, particularly as the World Health Organization predicts a significant rise in the number of individuals aged >65 years by 2050 [1]. To confront this impending public health challenge, the promotion of regular physical activity (PA) is paramount [2].

Research has consistently demonstrated the multifaceted advantages of regular PA on physical, cognitive, and mental health even in advanced age. In recognition of these benefits, the World Health Organization advocates for specific PA guidelines for individuals aged ≥65 years [3,4]. These guidelines encompass moderate-intensity aerobic activities, muscle-strengthening exercises, and balance training. However, despite the wealth of evidence supporting these recommendations, a substantial proportion of older adults worldwide fail to meet the prescribed PA levels [5,6]. Hence, adherence to recommended guidelines remains a challenging issue [7].

Addressing the gap in PA participation among older adults necessitates the implementation of innovative and sustainable interventions. Mobile technology has opened up avenues for innovative approaches to foster PA and cultivate healthier lifestyles even among older adults [8,9]. Mobile health (mHealth) apps have emerged as promising and cost-effective health intervention tools, especially in promoting PA. These apps harness technology to offer personalized guidance and track and encourage PA [10]. Capitalizing on the widespread use of smartphones and tablets, these apps possess the potential to revolutionize how we address and promote PA among aging populations [11]. They offer convenient access to PA advice and support, deliver an enjoyable user experience, and furnish feedback on progress over time [12-14].

Despite these positive trends, adoption of these technologies in real-life conditions is still relatively limited. Several challenges and hurdles must be overcome before such interventions can be successfully and sustainably implemented in the field. The challenge is not solely in developing mHealth apps but also in maintaining long-term engagement and motivation among older adults. In addition, studies often report high attrition rates and small intervention effects [14-16]. These problems must be addressed to use the full potential of mHealth interventions. First, high attrition is likely due to the intervention not matching the users’ needs, goals, and expectations [17,18]. This may be avoided by involving potential users during the entire cycle of intervention development [19-22]. Second, research has revealed that theory-based interventions are more effective at modifying health behaviors than traditional interventions [23,24]. Thus, interventions should be grounded within and informed by theoretical models.

### Objectives

In response to these challenges, a collaborative cocreation process led to the development and refinement of an mHealth app named More In Action (MIA) [19]. The app’s content and design were crafted through cocreative workshops and based on the theoretical framework of the Behavior Change Wheel (BCW) [23]. Despite the active involvement of end users from the outset of development and throughout the iterative process, as well as preliminary results indicating high levels of enjoyment and ease of use of the app [19], the long-term retention rate and motivation levels of the participants remain unknown. Therefore, the primary objective of this study was to thoroughly explore the acceptability, feasibility, and usability of the MIA app in promoting PA and encouraging sustained, active, and healthy behaviors among its users.

## Methods

### Study Design

**Figure 1:**
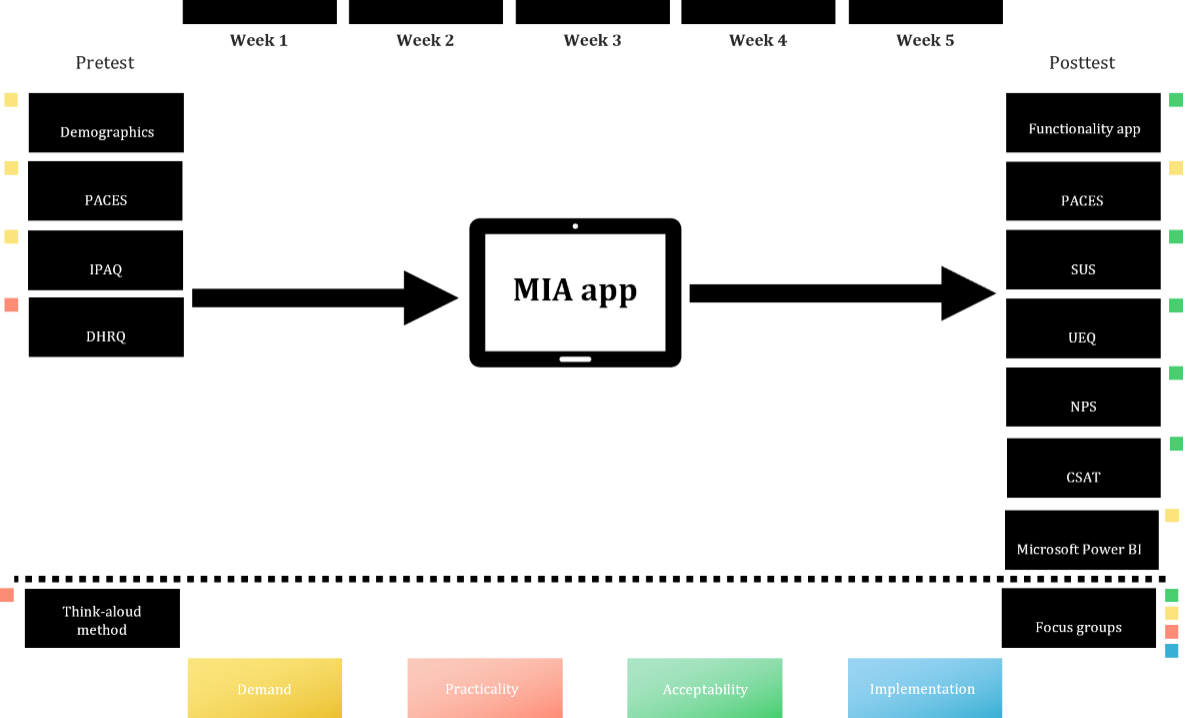
Study design. The colors represent the different evaluation focuses: yellow for demand (interest and need), orange for practicality (feasibility), green for acceptability (user satisfaction and acceptance), and blue for implementation (integration into the study). CSAT: Customer Satisfaction Score; DHRQ: Digital Health Readiness Questionnaire; IPAQ: International Physical Activity Questionnaire; MIA: More In Action; NPS: Net Promoter Score; PACES: Physical Activity Enjoyment Scale; SUS: System Usability Scale; UEQ: User Experience Questionnaire.

We used a comprehensive mixed methods approach to assess the MIA app’s acceptability, feasibility, and usability, as shown in Figure 1.

Our methodology adhered to the recommended framework for feasibility studies by Bowen et al [25]. This framework allowed us to explore critical aspects of the MIA app, such as *acceptability*, *demand*, *practicality*, and *implementation*. These interrelated concepts were systematically integrated into our study design and procedures, as detailed in Table 1.

Acceptability was defined as the perceived suitability of the app and is directly tied to user satisfaction and their intent to continue using the app, as shown in Table 1. Feasibility, on the other hand, addresses the ease of implementing the app among older adults [26], focusing on demand and implementation and examining whether it can be effectively implemented and sustained in users’ daily routines. In Table 1, feasibility is broken down into 2 distinct areas. The first is demand, which addresses whether older adults are willing to adopt and regularly use the app. This area evaluates actual use, intention to use, and perceived demand. The second is practicality, which assesses how easily the MIA app can be integrated into the users’ daily lives. This area was explored through the *think-aloud* method, focus groups, and questionnaires. Practicality measures the ease of integrating the app into daily routines, user willingness to pay, and any perceived positive or negative effects. Usability, a key aspect of our investigation, was defined as the ease of use and suitability of the system or product for a specific user group performing designated tasks in a particular environment, including practicality. In this context, *ease of use* directly impacted user performance and satisfaction, whereas *acceptability* determined the likelihood of the product being embraced and used [27]. In Table 1, usability directly impacts acceptability (satisfaction and intent to continue using the app) and feasibility (ease of integration and ability to perform the required intervention activities).

**Table 1:** Key areas of focus for the More In Action (MIA) feasibility study based on the framework by Bowen et al [25].

Area of focus and research question						Method			Outcome			Measures
**Usability**												
	**Acceptability**											
		To what extent is the MIA app as a means to promote PA^a^ acceptable among community-dwelling adults aged ≥65 years?			Questionnaire Focus group			Satisfaction Intent to continue use			SUS^b^, CSAT^c^, NPS^d^, and Likert-scale questions on how the app fits into the end users’ daily-life activities	
	**Feasibility**											
		**Demand**										
			What is the level of adoption of the MIA app among community-dwelling older adults? What factors influence the intention to use and engage with the app?	Questionnaire Microsoft Power BI (Microsoft Corp) analytics Plausible Analytics			Actual use Intention to use Perceived demand			Questions regarding the influence of the app on modulating PA behavior (eg, PACES^e^ and IPAQ^f^) and analytics to compare the frequency of use and patterns of use across the participants		
		**Practicality**										
			To what extent can the MIA app be integrated into the daily lives of older adults aged ≥65 years residing within the community?	Think-aloud method Questionnaire Focus group			Positive or negative effects Ability of participants to execute intervention activities Willingness to pay			First impressions, Likert scale, and in-depth questions regarding integrating the app into their daily lives (eg, DHRQ^g^)		
		**Implementation**										
			How can the MIA app be optimally implemented to facilitate sustained engagement in PA among community-dwelling older adults?	Focus group			Amount and type of resources needed to implement Factors affecting implementation			In-depth questions on how the MIA app can be deployed in the community context		

^a^PA: physical activity.

^b^SUS: System Usability Scale.

^c^CSAT: Customer Satisfaction Score.

^d^NPS: Net Promoter Score.

^e^PACES: Physical Activity Enjoyment Scale.

^f^IPAQ: International Physical Activity Questionnaire.

^g^DHRQ: Digital Health Readiness Questionnaire.

### Ethical Considerations

This study was registered at Clinical Trials.gov (NCT05650515) and was approved by the ethical committee of Hasselt University (B1152023000011). All participants provided informed consent. To ensure privacy, the data used in this study were deidentified before analysis. Participants did not receive any compensation for their involvement.

### Participants

Older adults without severe illness were invited to participate in this study. They were recruited via social media outreach, newspaper advertisements, and pitches at several older adult organizations and through the local community services during the recruitment period from August 2023 to September 2023. The inclusion criteria for participants encompassed that they had to be aged ≥65 years, competent to provide informed consent (Multimedia Appendix 1), able to actively participate in the study, community dwelling (living either independently at home or in a serviced apartment), without any severe illnesses, and native Dutch speakers. The exclusion criteria were the presence of current neurological, cardiovascular, respiratory, severe metabolic, or cognitive disorders. Participants were not excluded based on digital literacy, ensuring a diverse range of digital competence levels. The complete list of exclusion criteria is presented in Multimedia Appendix 2.

**Figure 2:**
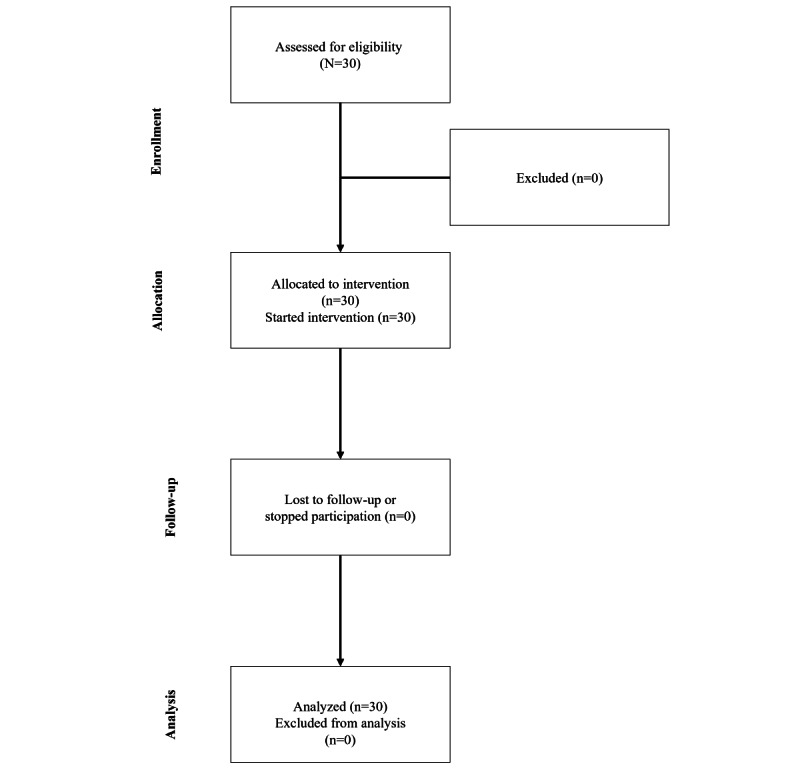
Modified CONSORT (Consolidated Standards of Reporting Trials) flow diagram for a single-arm feasibility study of the 5-week More In Action intervention.

Figure 2 provides a CONSORT (Consolidated Standards of Reporting Trials) flowchart for this single-arm feasibility study. The methodology and results were reported following the CONSORT 2010 checklist [28].

### Intervention

#### General Description

The mHealth app MIA represents an innovative approach to promoting PA and fostering a lifestyle centered on health and activity among older adults. Developed through a collaborative cocreation process, MIA has been refined over several iterations to meet the specific needs and preferences of its target user group [19]. MIA is optimized for both smartphone and tablet use, thereby ensuring accessibility and user engagement across a broad spectrum of mobile devices.

The app’s design and content draw upon insights gained from cocreative workshops and are firmly grounded in the intervention functions of the BCW theoretical framework [29] as well as principles from self-identification theory [30,31] as key elements to motivating individuals toward healthier behaviors. Textbox 1 presents a comprehensive summary of the behavior change techniques incorporated into MIA.

**Overview of behavior change techniques implemented in the More In Action (MIA) app.**

**Behavior Change Wheel (BCW) [32]**

- The BCW framework is centered on 3 core components: capability, opportunity, and motivation (Capability, Opportunity, and Motivation–Behavior system), which can drive behavior change. The MIA app enhances users’ *capability* through educational content and reminders, increases *opportunity* through social features such as the community calendar, and boosts *motivation* by offering personalized goals and feedback.

**Self-determination theory (SDT) [33]**

- SDT emphasizes 3 fundamental psychological needs—autonomy, competence, and relatedness—as essential for motivation toward healthy behavior changes:
- *Autonomy*: the MIA app can provide users with the ability to customize their exercise routines, choose their goals, and select the types of physical activity (PA) they prefer. The workouts are composed personally based on the tailored personal goals users enter after registering.
- *Competence*: to enhance users’ feelings of competence, the MIA app incorporates a system of progressive challenges and feedback. The app tracks users’ progress in real time, offering progress bars or celebratory messages for achieving milestones (eg, achieving the World Health Organization guideline for PA). In addition, educational content on the benefits of regular PA and other health benefits helps users feel more skilled and capable.
- *Relatedness*: the MIA app fosters a sense of relatedness by integrating a community calendar that allows users to connect with others.

**Self-identification theory (SIT) [31]**

- Implementing SIT within the MIA app involved creating features that allow older adults to integrate PA into their self-concept, making it a core part of their identity. Users start by *selecting goals* that resonate with their personal aspirations and lifestyle. These goals can range from improving health, gaining strength, and enhancing mobility to participating in community activities or playing with grandchildren. They choose freely. The key is for users to choose goals that reflect their values and how they perceive themselves.

#### Unique Attributes of MIA

The MIA app exhibits several unique attributes within the scope of gerontechnology that set it apart from other existing mHealth technologies. To compare MIA with existing solutions, we conducted a review to map the currently available mHealth technologies for promoting PA in older adults.

All technologies were scored according to 11 key components previously identified [19,20,34]. These findings are detailed in Table 2.

**Table 2:** Comparative analysis of mobile health app features for enhancing physical activity among older adults.

Existing apps	User-centered design^a^	Behavior change techniques^b^	Personalized intervention^c^	Interactivity^d^	Activities of daily living^e^	Integration with wearable devices^f^	Social cohesion^g^	Education and information^h^	Rewards and incentives^i^	Accessibility and inclusion^j^	Older adult–specific features^k^
StandingTall [35]			✓^l^	✓						✓	✓
Web + [36]		✓	✓			✓	✓	✓	✓	✓	✓
Ready Steady Go [37]		✓		✓			✓	✓	✓	✓	✓
HBex [24]	✓	✓	✓	✓	✓			✓	✓	✓	
Vivo [38]	✓			✓						✓	
App-based exercise program [39]			✓		✓			✓		✓	✓
Physitrack [40]			✓	✓						✓	
My plan 2.0 [41]	✓	✓	✓	✓				✓	✓	✓	✓
Bingocize [42]				✓			✓	✓	✓	✓	✓
Fit for All platform [43]			✓	✓					✓		
ActiveLifestyle [44]		✓	✓					✓		✓	✓
Vibrotactile app [45]	✓		✓	✓					✓		✓
PACE^m^ app [46]			✓		✓	✓			✓	✓	
Gymcentral [47]	✓	✓	✓	✓			✓		✓	✓	✓
Telehealth intervention [48]			✓	✓			✓				
Nymbl [49]			✓	✓				✓	✓		✓
eLIFE [50]		✓	✓	✓				✓	✓	✓	✓
Exercise app [51]			✓	✓					✓	✓	✓
Make Movement Your Mission [52]		✓		✓	✓		✓	✓	✓	✓	✓
Evident [53]		✓	✓	✓		✓		✓	✓		✓
MIA^n^ app [19]	✓	✓	✓	✓	✓	✓	✓	✓	✓	✓	✓

^a^This design prioritizes the end user’s needs, preferences, and limitations throughout the development process.

^b^These are systematic strategies derived from behavioral science theories to influence and sustain behavior modification.

^c^Personalized interventions involve tailoring health-related strategies and communications to individual users based on specific data gathered about their behaviors, preferences, and environmental contexts.

^d^Refers to the dynamic capability of the app to engage users through direct and responsive interactions.

^e^Integrates physical activity into routine daily tasks to reduce perceived barriers and enhance the practicality of exercises.

^f^This component involves the app’s capability to synchronize with wearable technology to gather continuous physiological data, which can be used for monitoring health conditions in real time.

^g^Encourages the formation of supportive social networks within the app, enhancing user engagement through community building.

^h^Delivers evidence-based health information and instructional content to improve knowledge and skills related to physical activity.

^i^Uses motivational elements such as web-based badges, achievement unlocking, and progress tracking to enhance motivation and encourage continual app engagement.

^j^Accessibility ensures that products and services are usable by people with various abilities, whereas inclusivity focuses on creating environments that accommodate and welcome diverse individuals across all backgrounds and needs.

^k^An older adult–specific feature is a design element in a product or service, particularly mobile apps, that addresses the physical, cognitive, and social needs of older adults, such as mobility, sensory impairments, chronic conditions, and isolation, to enhance usability, safety, and autonomy.

^l^Presence of component.

^m^PACE: Physical Activity Cardiorespiratory Exercise.

^n^MIA: More In Action.

#### Features

MIA incorporates 7 major features: tailor-made tips, literacy initiatives to enhance awareness about PA and a healthy lifestyle, guided exercise workouts with peers, a community calendar fostering social connections, a personal diary for manual uploads of non–app-based PAs, a progression monitor, and a chatbot named MIA. The chatbot serves as a platform where users can pose questions and receive insights, motivation, and support in return. The current chatbot function is managed manually, with researchers on the development team directly addressing user inquiries. This approach allows for personalized responses and ensures that user concerns are addressed with accuracy and expertise but also for the collection of data that will be used later on to develop an artificial intelligence (AI)–driven chatbot. This AI chatbot would maintain human oversight to ensure the accuracy and relevance of the responses, particularly for complex queries. The app also includes 195 guided workout videos, each featuring 30-second exercise bursts followed by breaks. These exercises cover strength, endurance, coordination, and balance and are demonstrated by peers. Workouts are tailored to 3 skill levels—beginner, advanced, and expert—and range from 10 to 20 minutes. MIA continuously adapts workouts based on user experiences; for example, if an exercise is too challenging, the algorithm adjusts future sessions to ensure that they remain enjoyable and achievable.

#### User Onboarding and Interaction

Upon initiating MIA, users undergo a preliminary assessment through a questionnaire that informs the customization of their exercise regimen. This personalization is central to MIA’s approach, tailoring the app experience to individual needs and preferences.

**Figure 3:**
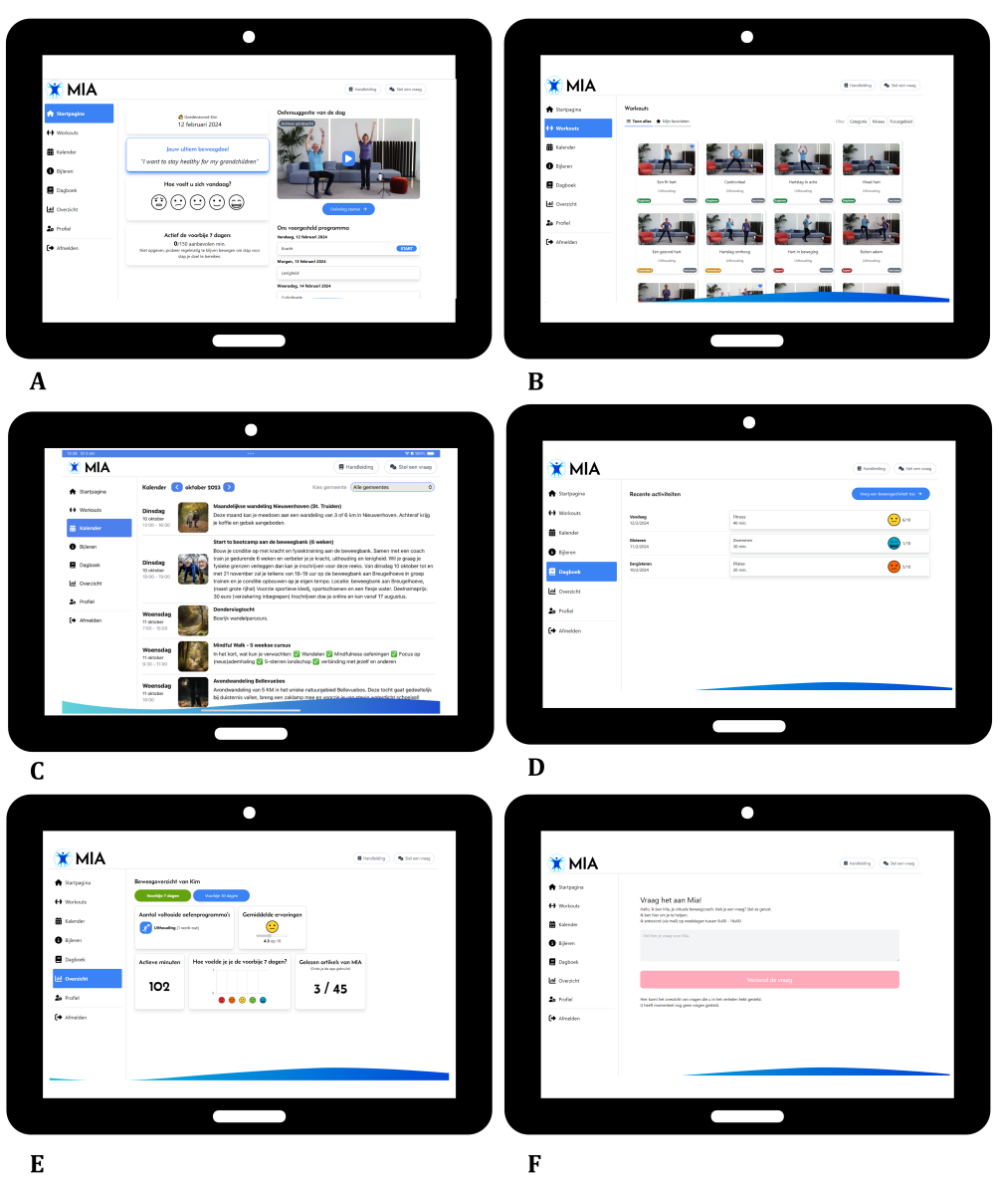
Screenshots of the More In Action interface: (A) home page displaying a physical activity (PA) agenda, suggested workout videos, and daily tips designed to engage users with personalized fitness guidance; (B) library of workout videos with filters for exercise type, fitness level, and target body areas supporting a tailored workout experience; (C) a community calendar for local PA events; (D) manual logging of nonapp PAs; (E) PA progress monitor; and (F) a web-based coach. MIA: More In Action.

As shown in Figure 3, the home page presents a personalized PA agenda that adapts daily based on user feedback to align with their physical and mental health status. This adaptive feature is critical for tailoring the experience to the user’s day-to-day condition, emphasizing the interconnection between mental well-being and PA.

Throughout the intervention period, participants were not required to use the app daily. Instead, following the principles of behavior change techniques, autonomy was emphasized, allowing users to set personalized goals and choose their own frequency of app engagement. This flexibility enabled participants to engage with the app at their preferred rate, from as little as once a week to daily over the course of the 5-week intervention.

### Outcome Measures

#### Quantitative Measures

#### At Baseline

Demographics and PA levels were first assessed during the baseline measurement. The International Physical Activity Questionnaire–Short Form (IPAQ-SF) [54], the Physical Activity Enjoyment Scale (PACES) [55], and the Digital Health Readiness Questionnaire (DHRQ) [56] were used to measure digital health readiness via a web-based questionnaire using Qualtrics (Qualtrics International Inc). The IPAQ-SF showed moderate validity with accelerometry but had wide limits of agreement, indicating caution for longitudinal use [57,58]. Both the PACES [55,59] and DHRQ [56] demonstrated strong internal consistency and reliability, making them suitable for assessing enjoyment of PA and digital health readiness, respectively.

#### The 5-Week Postintervention Assessment

To evaluate user satisfaction and experience, 3 established instruments were used: the System Usability Scale (SUS) [60], Net Promoter Score (NPS) [61], and Customer Satisfaction Score (CSAT) [62]. The SUS provides a standardized assessment of usability through 10 items with response options ranging from *Strongly agree* to *Strongly disagree*. Scores of >68 indicate above-average usability. The SUS has demonstrated high internal consistency (Cronbach α=0.74) and test-retest reliability (Pearson correlation coefficient=0.75), supporting its use in evaluating digital health interventions [63]. The NPS measures user loyalty by asking participants to rate their likelihood of recommending the app on a scale from 0 to 10. This metric is calculated by asking older adults the following: *On a scale from 0 to 10, how likely are you to recommend MIA-app to a friend or peer?* Respondents are classified into 3 categories: promoters (those who provide a score of 9-10), passives (those who provide a score of 7-8), and detractors (those who provide a score of 0-6). The NPS, derived by subtracting the percentage of detractors from that of promoters, offers a straightforward approach to understanding user loyalty and referral potential [62].

The CSAT quantifies user satisfaction using a 10-point scale, capturing immediate feedback on the app experience. App-specific concerns (user-friendliness, layout, and utility) were also evaluated across multiple categories, with ratings ranging from 1 to 5 (1-2 indicating dissatisfaction, 3 indicating neutrality, and 4-5 indicating satisfaction).

Microsoft Power BI (Microsoft Corp) was used to analyze participant behavior during app use to provide insights into app use analytics and user engagement patterns [64]. Data on metrics such as workout frequency, session duration, and instances of premature workout termination were collected using integrated tracking tools such as Plausible Analytics. These data were imported into Microsoft Power BI for further analysis. Web-based dashboards and visualizations, including bar charts, were created to represent key engagement metrics and user navigation patterns.

#### Qualitative Measures

#### At Baseline

A *concurrent think-aloud* approach allowed participants to voice their thoughts and actions while using the app throughout installation and use [65,66]. Usability and enhancement suggestions were supplied to the researchers in real time [67-69]. Multimedia Appendix 3 details the think-aloud protocol, with an average procedure time of 20 (SD 6.2) minutes. These sessions were conducted in our laboratory at PXL University of Applied Sciences and Arts.

#### The 5-Week Postintervention Assessment

Qualitative data from focus groups were also collected to better understand app users’ experiences. Following the 5-week trial of the app, the participants were invited to take part in focus groups in our laboratory at PXL University of Applied Sciences and Arts, each consisting of 6 older adults. Each focus group session lasted 2 hours and aimed to explore participants’ perceptions of the app along with their reflections and suggestions for improvement. The sessions covered all app features. The focus groups were facilitated by experienced researchers (JR and KD) who are specialized in qualitative research. Multimedia Appendix 4 contains the interview guide.

### Data Analysis

#### Quantitative Analysis

The quantitative analysis used mostly descriptive statistics to examine the data. Frequency distributions were also used to observe the occurrence of data values or categories, offering a detailed view of data distribution across outcomes. In addition, Microsoft Power BI analytics played a crucial role in visualizing and interpreting the data. Microsoft Power BI allowed for dynamic dashboards and visualizations, making it easier to explore trends, patterns, and relationships within the data. By integrating various data sources, it enabled the creation of charts, graphs, and tables that provided real-time insights into participant behavior and app use.

#### Qualitative Analysis

The think-aloud method and focus groups were audio recorded with participants’ consent for the qualitative component. The transcriptions of the focus groups were carried out verbatim, followed by a rigorous content analysis using the NVivo software (QSR International) [70]. Themes were systematically coded based on the guidelines by Braun and Clarke [71]. The coding process was independently carried out by 2 experienced researchers (KD and JR), ensuring reliability and reducing the potential for bias in theme interpretation.

#### Integration of Qualitative and Quantitative Results

The Nonadoption, Abandonment, Scale-up, Spread, and Sustainability (NASSS) framework was used to integrate both the qualitative and quantitative results of the study, providing a structured analysis of the potential for sustainable implementation of the MIA app [72]. Recognized for its utility in evaluating complex technological health interventions, the NASSS framework allows for a systematic examination of key factors influencing the deployment and long-term sustainability of digital health technologies. The analysis encompassed the framework’s 7 domains: the condition being addressed (physical inactivity among older adults), the technological characteristics of the MIA app (including usability and technical robustness), the value proposition (perceived benefits to end users and stakeholders), the adopter system (end users and supporters), the organizational context (integration within health care systems), the wider sociopolitical environment (regulatory and cultural considerations), and the processes of embedding and adapting the technology over time.

## Results

### Demographic Characteristics

A total of 30 participants (mean age 70.3, SD 4.8 y; n=17, 57% female) were included in this study. Participants had different educational backgrounds; the largest proportion (15/30, 50%) had completed higher education. Their level of digital literacy was commendable, displaying a mean score of 59.6 (SD 8.8) out of 75 on the DHRQ. Notably, 80% (24/30) of the participants acknowledged the affirmative impact of digitalization on health, whereas 73% (22/30) actively engaged with social media, among whom 36% (8/22) used it daily, 23% (5/22) used it often, 23% (5/22) used it occasionally, and 14% (3/22) used it rarely. Furthermore, 67% (20/30) of the participants sought health-related information on the web, whereas all 30 participants reported internet use, with 27 (90%) of them accessing it daily, and 17 (57%) owned a smartwatch. When it came to monitoring PA through their smartphones’ health apps, 73% (22/30) of the participants checked their step count daily, 13% (4/30) did so frequently, 3% (1/30) did so occasionally, 3% (1/30) did so rarely, and 10% (3/30) never did. Regarding health-related app use, 17% (5/30) of the participants actively used this type of apps.

The IPAQ-SF revealed variability in PA levels among individuals. The median activity levels indicated moderate engagement in walking (1386, IQR 284.8-2178 metabolic equivalent of task min/wk) and lighter involvement in vigorous activities (median 0, IQR 0-1830), suggesting a skew toward lower-intensity exercises. Moreover, it was found that 10% (3/30) of the participants were classified as inactive, 40% (12/30) fell into the category of minimal activity, and a significant 50% (15/30) were categorized as vigorously active. High SDs across all categories underscored a wide range of PA levels, from minimal to extremely active, reflecting the diverse nature of physical engagement in this sample.

The PACES results revealed that participants, on average, reported a moderate to high level of enjoyment during PA, with a mean score of 107 [55]. Considering that the PACES scale ranges from 18 to 126, where higher values signify greater enjoyment, the mean score of 107 suggests that participants’ enjoyment levels were significantly skewed toward the upper end of the scale. This positioning implies a generally positive perception of PA among the participants. The complete sociodemographic characteristics of the participants are presented in Table 3.

**Table 3:** Participant characteristics (N=30).

Variable			Values
**Age (y), mean (SD)**			70.3 (4.8)
**Sex (female), n (%)**			17 (57)
**Marital status, n (%)**			
	Single	2 (7)	
	Living together	1 (3)	
	Married	21 (70)	
	Divorced	4 (13)	
	Widowed	2 (7)	
**Educational level, n (%)**			
	Primary school	1 (3)	
	Middle school	6 (20)	
	University of applied sciences	15 (50)	
	University	8 (27)	
**Fall incidence (yes), n (%)**			3 (10)
**Digital literacy score (DHRQ** ^a^ **; out of 75), mean (SD)**			59.6 (8.8)
	Use (out of 20)	16.3 (3.1)	
	Skills (out of 25)	21.3 (3.2)	
	Literacy (out of 15)	12.3 (2.2)	
	Health literacy (out of 15)	9.8 (3.2)	
	Learnability (out of 25)	20.7 (2.5)	
**Physical activity level (IPAQ** **-** **SF** ^b^ **), median (IQR)**			
	Total	3273 (1345.5-3873)	
	Walking	1386 (284.8-2178)	
	Moderate	1020 (310-2070)	
	Vigorous	0 (0-1830)	

^a^DHRQ: Digital Health Readiness Questionnaire.

^b^IPAQ-SF: International Physical Activity Questionnaire–Short Form.

### Quantitative Analysis

#### Acceptability

**Figure 4:**
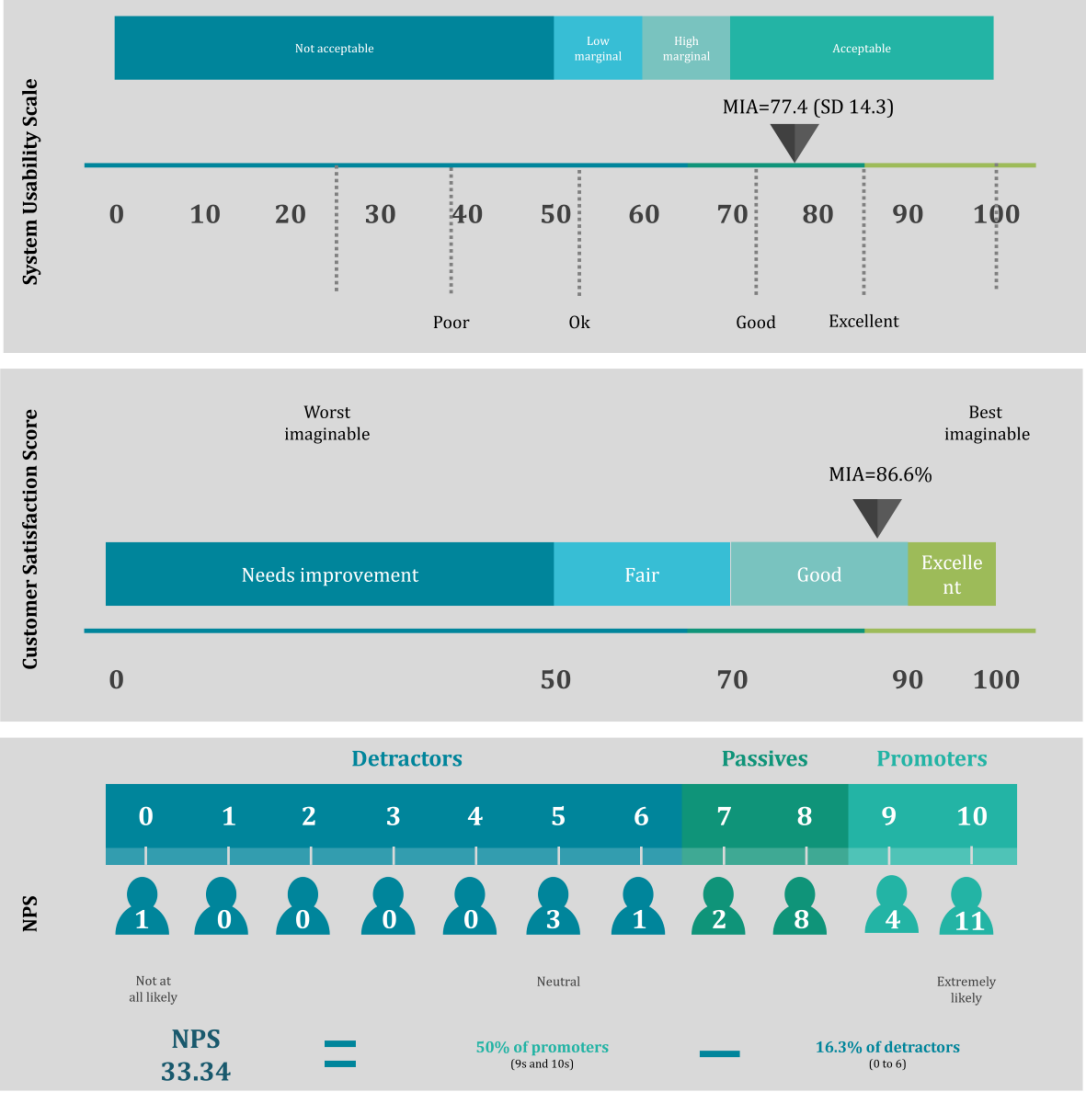
Results of the System Usability Scale, Customer Satisfaction Score, and Net Promoter Score (NPS). MIA: More In Action.

First, the general acceptability was accessed using the 3 main indicators, as shown in Figure 4.

According to the SUS, the mHealth app was considered *acceptably good*, with a mean score of 77.4 (SD 14.3). The results of the CSAT revealed that 47% (14/30) of the participants indicated that they were *very satisfied*, whereas 40% (12/30) reported being *satisfied*. Another 10% (3/30) had a neutral response, and only 3% (1/30) were *dissatisfied*. None of the participants provided a *very dissatisfied* score. This distribution led to a CSAT score of 86.6%, indicating a high satisfaction level among older adults using the MIA app. The NPS was used to assess customer satisfaction with and loyalty and enthusiasm toward the app. A total of 50% (15/30) of the participants were *promoters*, indicating that they would recommend the MIA app to peers and family. In addition, 33% (10/30) were *passives*, reflecting moderate satisfaction without strong advocacy, whereas 17% (5/30) were *detractors*, indicating that they were unlikely to recommend the app. The resulting NPS is calculated by subtracting the percentage of detractors from the percentage of promoters, yielding a total NPS of 33.34.

**Figure 5:**
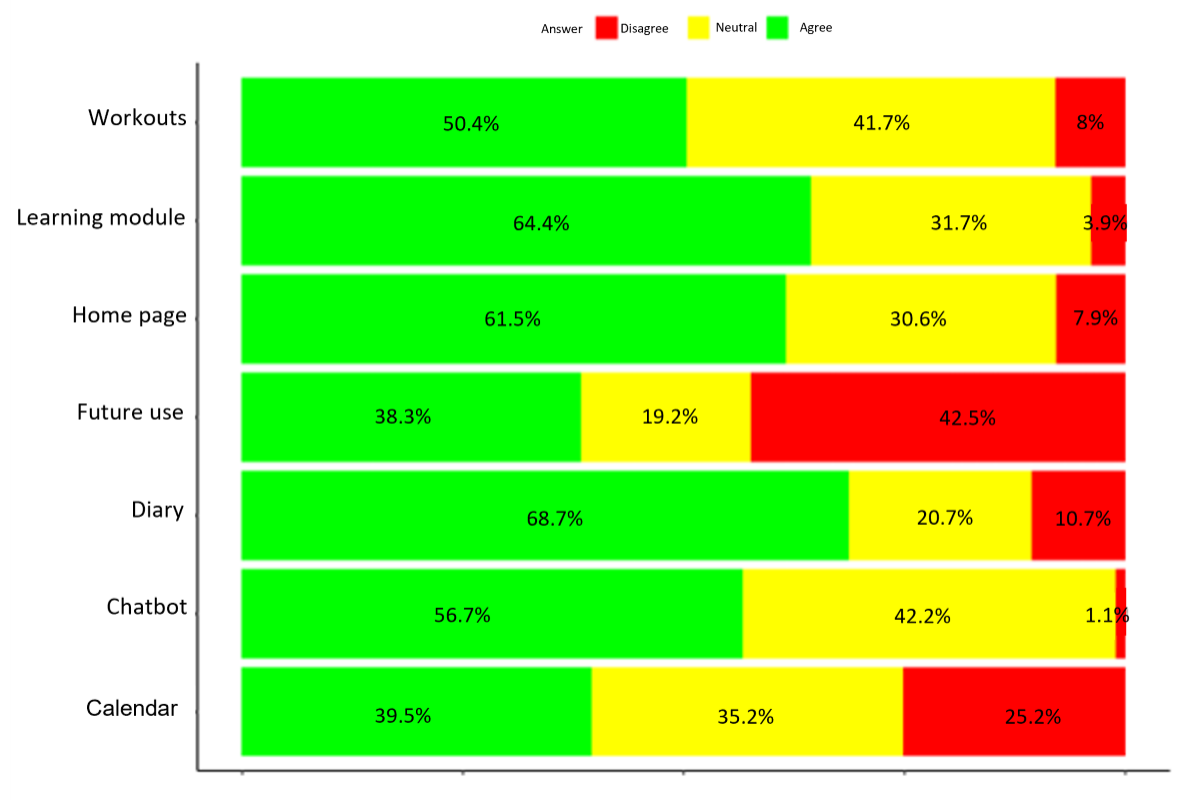
User satisfaction and engagement—categorized ratings of app features and interface (1-2: dissatisfaction [red]; 3: neutrality [yellow]; 4-5: satisfaction [green]).

Furthermore, the specific acceptability of the different features was assessed. The quantitative results also guided the qualitative part of the study, where focus groups were conducted to dive deeper into participants’ reflections and gather additional insights. The aggregated results are presented in Figure 5. The complete results per category are presented in Multimedia Appendix 5.

#### User Behavioral Patterns

To track user navigation and behavior within the app, Microsoft Power BI analytics were collected on the following features: workout videos, learning modules, community calendar, manual diary, progression monitor, and chatbot. Visual representations of the Microsoft Power BI analytics are available in Multimedia Appendix 6.

#### Workout Videos

A total of 585 workout videos were analyzed, highlighting general satisfaction with a median satisfaction score of 4 (IQR 3-4; scale of 1-5) and physical exertion rated at a median of 5 (IQR 5-7; scale of 0-10). Users engaged for a median duration of 14 (IQR 0-34) minutes per session, with use times ranging from 0 to 34 minutes. Strength workouts were most common, comprising 40% (234/585) of the sessions, followed by endurance, flexibility, and balance workouts. Most workouts (429/585, 73.3%) were aimed at beginners, with only 5% (29/585) targeting expert levels, reflecting the app’s focus on older adults. Notably, 77% (23/30) of users expressed no preference for a specific type of exercise, suggesting a wide acceptance of the available workout options.

#### Learning Modules

The distribution of the learning modules comprised 255 read articles across 6 different topics: mental health, social well-being, physical well-being, nutrition, sleep, and risk of falling. Physical well-being was the most frequently engaged with topic, with 26.3% (67/255) of the total read articles. Nutrition followed with 17.3% (44/255) of the read articles, whereas mental health comprised 16.9% (43/255) of the read articles.

#### Community Calendar

Only 3 activities were added to the participants’ community agenda, all of which were organized walks.

#### Manual Entries in Diary

The analysis of 545 external activities showed a median *general feeling after exercise* score of 4 (IQR 4-4) on a scale from 2 to 5, indicating generally positive responses. The range of perceived exertion scores was broader, with a median of 5 (IQR 5-7) indicating a balance between lower and higher levels of perceived effort. Walking emerged as the most popular activity (210/545, 38.5% of external activities), followed by cycling with electric assistance (78/545, 14.3%); cycling without electric assistance (66/545, 12.1%); and other activities such as padel, tennis, strength training, and running. The duration of these activities varied significantly, with a median time of 60 (IQR 40-90) minutes ranging from 5 to 400 minutes, indicating diverse engagement levels. The data revealed that a quarter of the participants (8/30, 27%) engaged in activities for <40 minutes, whereas a significant portion spent ≥90 minutes.

#### Chatbot

During the 5-week period, the chatbot was consulted 66 times. Most of the interactions involved feedback, accounting for 48% (32/66) of instances. Communication regarding exercise differentiation occurred 15% (10/66) of times, whereas medical-related queries were raised 5% (3/66) of times. Technical issues led to 9% (6/66) of consultations, and motivational support prompted 17% (11/66) of interactions. The remaining 6% (4/66) of consultations fell into other categories.

#### Adherence

During the 5-week intervention, app adherence was measured by tracking both the frequency of use and interaction patterns. Rather than requiring daily use, the study sought to assess whether participants engaged with the app according to the personalized goals and self-determined use frequencies they had set. The intervention, guided by self-determination theory [33], prioritized autonomy, enabling participants to select their own goals and decide how often to use the app. Engagement levels varied, with participants using the app between 3 and 7 times per week, with a median of 5 (IQR 4-6) times per week. Adherence rates averaged 135%, with individual rates ranging from 21% to 271%, reflecting the diverse levels of user interaction throughout the intervention.

On average, participants engaged with the app 32.88 (SD 14.06) times, ranging from 11 to 59 times used, which translates to an approximate daily use rate of 0.94 (SD 0.40) times. This indicates near-daily use of the app among the study cohort.

**Figure 6:**
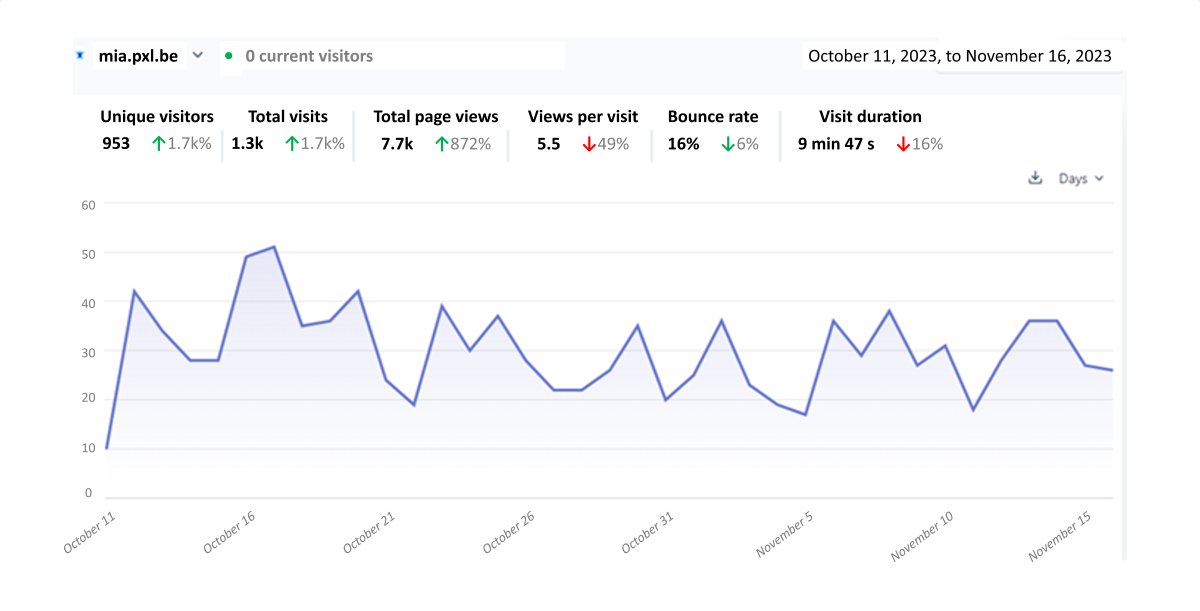
Participant engagement and interaction patterns over the 5-week intervention. The y-axis shows the number of visits to the More In Action app.

Despite the considerable variability in workout interactions within the app, with a median workout frequency of 17 sessions (IQR 11.2-28.5), motivation remained notably stable throughout the study, with some dips during the weekends. Participant engagement and interaction patterns throughout the 5-week intervention are visualized in Figure 6. Daily engagement varied between 0.66 and 3 sessions per day; however, there were no discernible fluctuations in adherence over time. Unlike the common pattern of an initial surge in activity followed by a dip and potential recovery, participants demonstrated consistent interaction with the app across the 5-week intervention.

### Qualitative Analysis

#### Think-Aloud Method

The thematic analysis of the *think aloud* data resulted in the identification of 3 major key themes presented in Textbox 2. The other results and functionality are briefly described in the following sections.

Participants began interacting with the MIA app by opening it and completing their profiles. The welcome screen was inviting and easy to navigate, but some participants encountered difficulties with the profile questionnaire, highlighting a need for clearer guidance. When exploring the home screen, the interface was straightforward, with large icons and a simple layout. However, some features such as mood indicators were confusing, indicating a need for better explanations. The workout section was well received for its variety and suitability for different fitness levels, enhancing user experience. However, navigating the calendar feature proved challenging for some, with issues in adding or removing items pointing to a need for a more intuitive design. The learning module page was appreciated for its informative content, although some noted a lack of retirement-related topics, suggesting room for expansion. The diary functionality faced some hurdles as users found it difficult to add past activities, needing more flexible controls. Visually, the app was praised for its appealing design with bright colors and clear typography, making it esthetically pleasing and not overly complex. However, specific challenges such as difficulties with the profile questionnaire and mood indicators revealed that clarity in guidance is also crucial. Thus, while the app’s visual appeal contributes to its overall usability, the complexity of certain features points to the need for balancing esthetics with intuitive guidance.

**Key themes of the think-aloud protocol.**

**Usability and accessibility**

- The participants’ experiences reveal that usability and accessibility are crucial for a successful app. Challenges with completing profiles, navigating calendars, and adding diary entries highlight areas in which the app could improve. Ensuring that the app is intuitive and easy to use can lead to a better user experience.

**User interface and design**

- The overall design and layout of the app play a significant role in user satisfaction. The positive feedback on the app’s simplicity, large icons, and bright colors shows that a visually appealing and straightforward design enhances usability. This theme indicates that maintaining a clean and user-friendly interface is essential.

**Feature functionality and clarity**

- The confusion regarding certain features, such as the mood indicators and the calendar, suggests that functionality and clarity are important. Ensuring that each feature is well explained and operates smoothly can lead to a more satisfying user experience. This theme points to the need for clear communication and improved design in specific areas of the app.

#### Focus Groups

#### Overview

**Figure 7:**
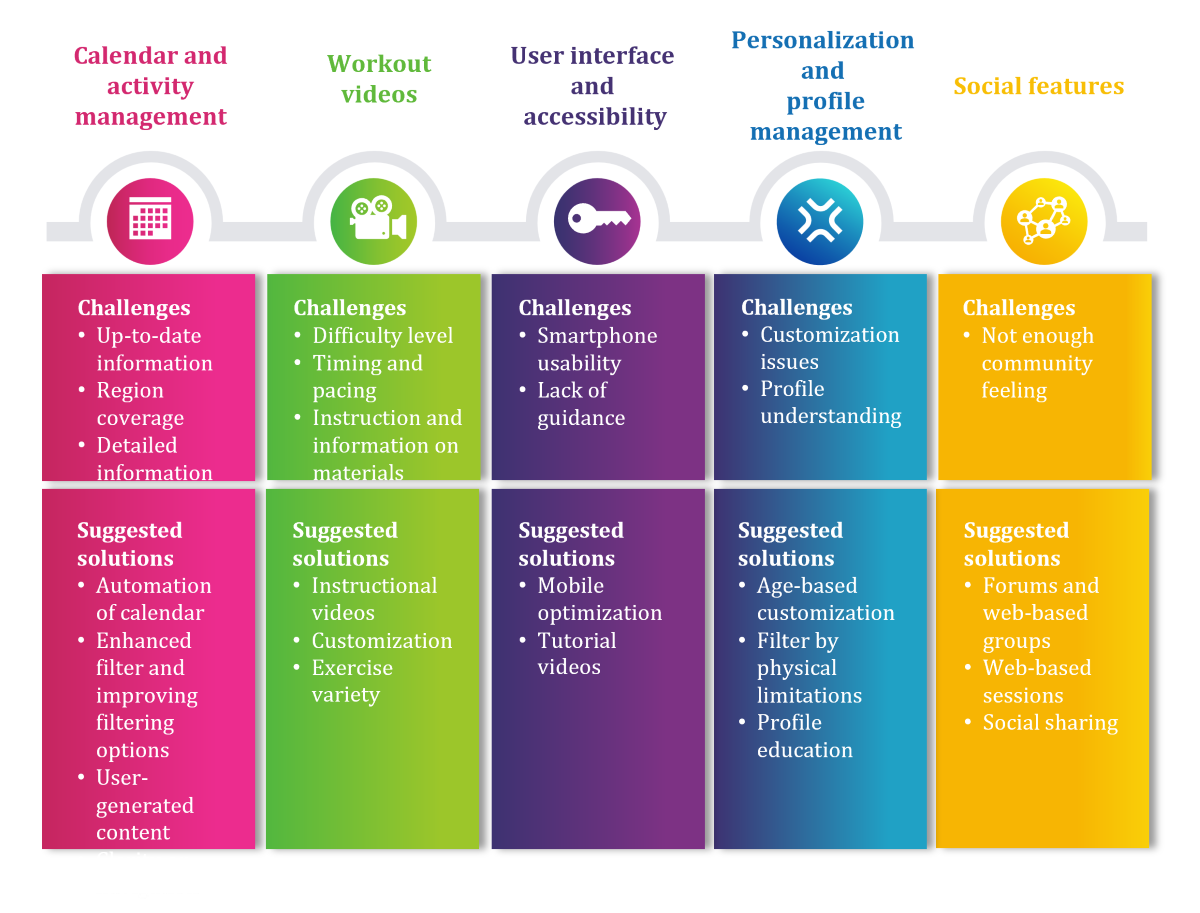
Key themes of the postassessment focus groups.

The transcripts of these focus groups led to identifying 5 key themes, presented in Figure 7: calendar and activity management, workout videos, user interface and accessibility, personalization and profile management, and social features. Participants valued the community calendar for fostering social connections, but engagement in activities was low. Tailored workout videos were appreciated for their adaptability, although more flexibility in goal setting and detailed instructions were desired. Feedback highlighted the intuitive interface but suggested improvements in flexibility, readability, and tracking. Personalization features such as the chatbot were appreciated, although users wanted better clarity in activity plan customization. Social features, including a buddy system, were seen as ways to boost user interaction and engagement. Each of these themes is further detailed in the following subsections.

#### Calendar and Activity Management

Participants valued the community calendar for enhancing social connections but reported limited participation in the listed activities. A participant aged 68 years highlighted the following:

The agenda is there because of the social connection with others. I really liked it, although I didn’t go to an organized activity. You should definitely keep it in there.

Others suggested that participation might increase with better visibility and advertising of the activities, along with reminders and notifications to prompt more active involvement.

#### Workout Videos

The tailored workout plans and videos were popular among users. A participant aged 67 years wanted greater control over his workout goals, saying the following:

The target of 150 minutes per week is good, but I would like to choose my target minutes myself, for instance, 500 minutes.

This comment indicated that some users prefer more flexibility in setting personal goals. Another participant aged 72 years commented the following:

The algorithm when a workout is too challenging works well; the next video suggestion is indeed tailored to my level. I tested it to see if it would work.

This adaptability was valued, but users pointed out that the video instructions could sometimes be too quick to follow. Another participant aged 70 years who was already active and doing a lot of cardiovascular exercise mentioned the following—“The app and the exercises made me realize I need to work on my strength as well”—suggesting that the app can broaden users’ fitness horizons.

Participants also suggested improvements such as being informed about required materials in advance. A participant aged 69 years noted the following:

It would be good to know which materials to use in advance.

This feedback suggests that clearer preparation instructions could enhance user experience.

#### User Interface and Accessibility

Various aspects of the app’s interface and accessibility were highlighted, noting strengths and areas for improvement. The calendar feature was appreciated for fostering social interactions, although it lacked sufficient engagement tools. Users found the manual diary and progression monitor helpful for tracking activities. Still, they criticized their limited functionality, particularly the inability to edit past entries and the short view range of 7 days. Suggestions included enhancing flexibility and extending tracking capabilities to improve usability.

The *Learning* section received mixed reviews; while the content was engaging, users suggested features such as checkboxes to track articles read, indicating a need for more interaction and relevance to older adults. Although the app’s layout was praised for its esthetic and ease of navigation, calls for better readability and customization were prominent.

Feedback on the chatbot was mixed, with some users finding it motivating whereas others saw room for personalization. General usability issues related to smartphone optimization and intuitive navigation were raised but reportedly resolved quickly, demonstrating effective technical support. While the app was generally well received, users desired more robust features and personalization to enhance their experience.

#### Personalization and Profile Management

Users appreciated features that allowed for personalization, such as the chatbot and manual diary, recognizing their value despite some limitations. A user aged 68 years praised the diary feature, stating that “The diary was great. It is really good to track your activities,” but also recommended enhancements, suggesting that “It would be nice if the diary could go back in the past, more than now.” In addition, it became apparent that users were unaware that the initial registration questions were intended to personalize their plans as a user stated the following:

I didn’t know we could adjust the information in the PA plan.

This misunderstanding also led to them not expecting the system to adapt over time.

Nonetheless, several participants acknowledged the app’s added value in promoting PA. A participant aged 66 years noted the following:

Getting the push notifications motivated me; it was like a digital motivator in being more active.

#### Social Features

The social features such as the community calendar were highlighted as significant opportunities to enhance user engagement. A participant aged 66 years suggested a buddy system to increase interaction:

Perhaps a buddy system where I can see my friend and we can motivate each other.

#### Suggestions for Improvement

Multimedia Appendix 7 summarizes the suggestions and iterations from the focus groups, offering insights into lessons learned during the development process.

### Integration of Quantitative and Qualitative Analysis

**Figure 8:**
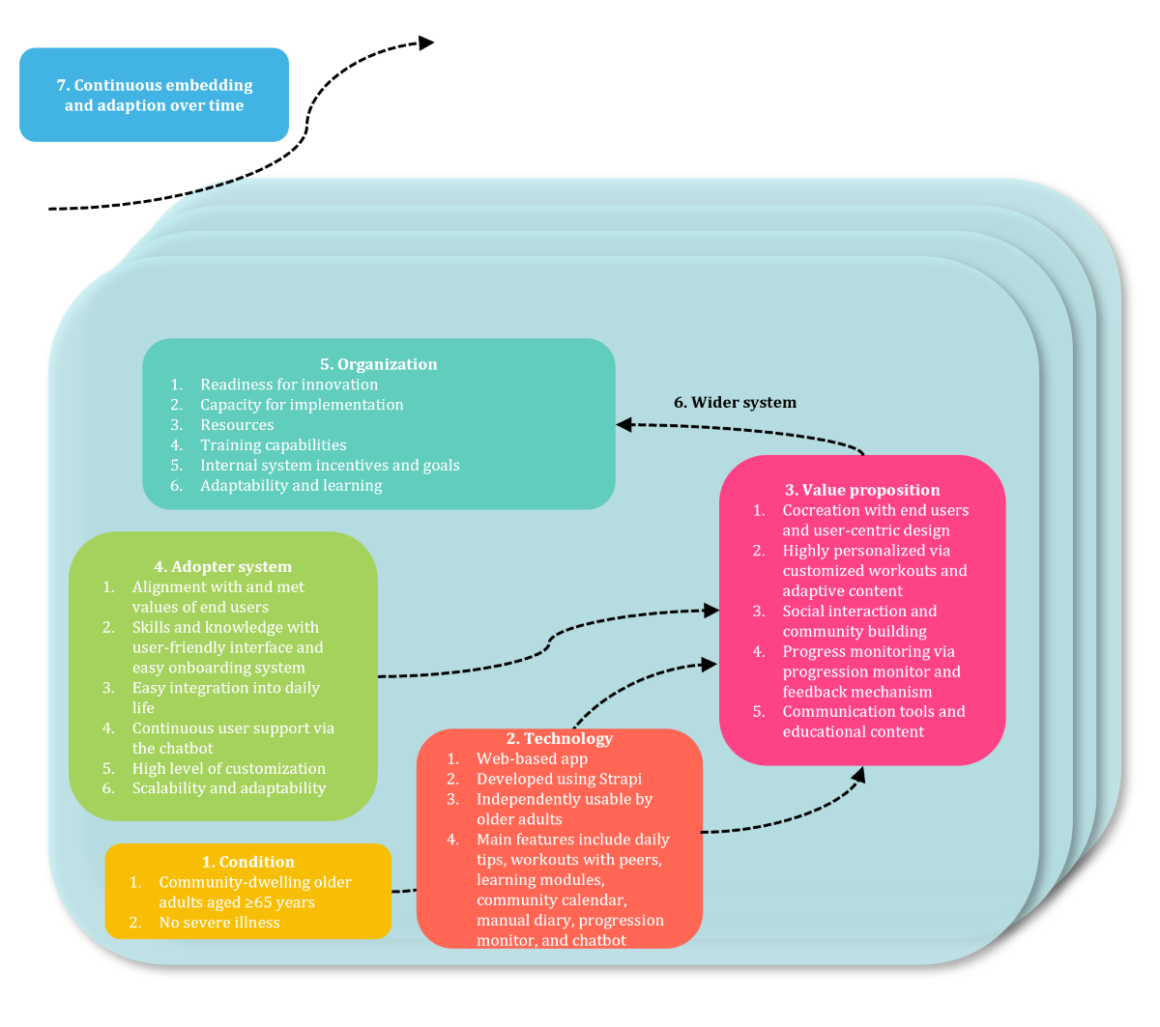
Nonadoption, Abandonment, Scale-up, Spread, and Sustainability framework adapted for the feasibility study of the More In Action app.

The NASSS framework [72] provided a structured approach to integrating the quantitative and qualitative results, revealing barriers to and facilitators of adoption and the interactions influencing scalability and sustainability. It enabled comparisons among user experiences, usability scores (eg, SUS and CSAT), and app interaction data while incorporating qualitative insights from focus group feedback on usability challenges and user perceptions. This enabled us to identify alignment and discrepancies between users’ perceived value of the MIA app and their actual app interactions, as well as broader contextual factors influencing sustained use. Figure 8 visualizes this analysis, emphasizing how the NASSS framework bridges the gap between numerical data and user narratives.

Addressing physical inactivity among older adults is challenging due to varying digital literacy, physical abilities, and health conditions. A key facilitator was the app’s customization, allowing users to adjust exercise recommendations, leading to positive SUS and CSAT scores. The app’s technical stability and responsive support further encouraged adoption among digitally literate users.

However, barriers such as navigation difficulties and insufficient guidance on features such as profile setup and calendar use limited engagement, with 30% (9/30) of the users classified as passive and 17% (5/30) classified as detractors in the NPS, indicating a risk of user churn. While users appreciated tailored workouts and real-time feedback, some sought greater control over goal setting, suggesting the need for enhanced personalization.

The NASSS analysis identified opportunities to boost engagement, including social features such as a buddy system to align with older adults’ social preferences. Integration with health care systems could facilitate broader adoption if endorsed by providers. Alignment with health policies and partnerships may also expand the app’s reach.

Despite these strengths, challenges such as technological intimidation, market competition, age-related declines, and data privacy concerns pose risks to long-term adoption. Adherence to ethical standards mitigated some concerns. Sustained engagement depended on the app’s adaptability to user feedback, requiring continuous updates and technical support to maintain user interest. Detailed information is available in Multimedia Appendix 8.

## Discussion

### Principal Findings

In recent years, the intersection of aging and technology has gained recognition as a vital factor in enhancing quality of life for older adults [73]. Technology holds substantial potential to improve everyday living for this demographic, yet adoption barriers remain a significant concern as outcomes often rely on user-driven interactions, which can vary significantly [10,34,74,75]. Effective technology design for older users is best achieved through *user involvement and cocreation*, a strategy that leverages the unique insights of users, who are experts in their own experiences [19,20,61,76,77]. This participatory approach not only results in products that better meet older adults’ specific needs, desires, and challenges but also counters age-related stereotypes in gerontechnology [78]. MIA exemplifies this by having been developed through a collaborative cocreation process with older adults. However, to achieve acceptable technologies for older adults, an in-depth understanding of their acceptability, feasibility, and usability is necessary [79]. Against this backdrop, the main aim of this study was to thoroughly explore the acceptability, feasibility, and usability of the MIA app in promoting PA and encouraging sustained, active, and healthy behaviors among its users.

The app received positive feedback, achieving an SUS score of 77.4 and a high CSAT rate of 86.6%. The NPS stood at 33.34, indicating a good level of customer loyalty, with half (15/30, 50%) of the participants categorized as promoters. Feedback on the app was positive for its clarity and engagement. In addition, feasibility and adherence levels were comparable to those observed in other apps evaluated. For example, StandingTall showed acceptable usability, with exercise adherence improving over time [35], whereas Physitrack reported a high study retention rate of 95% and an adherence to prescribed exercises of 84% [40]. Similarly, Fit for All [43] recorded an adherence rate of 82%, supporting the app’s potential in facilitating engagement and promoting PA among users. The workout videos on the MIA app received a commendation, although some participants suggested improvements. The learning module and diary functions were praised for their utility, yet some users noted challenges with navigation and desired the ability to edit past diary entries. The progression tracker and chatbot were also seen as valuable, although there was a call for more detailed tracking and enhanced personalization. Analysis showed strong engagement with the workout videos, especially strength-focused sessions, and high satisfaction levels with these workouts. Insights from the think-aloud protocols and focus groups highlighted the need for clearer guidance on profile management, workout customization, and calendar functionality. Participants also expressed a desire for more engaging social features, such as a buddy system. In general, the app was well received for its educational content and tracking features. However, there were recommendations for improving the user interface, enhancing social connectivity, and further personalizing the content.

There are 3 reasons why the MIA app offers more value compared to other alternatives (Table 2). First, the development and design process of the MIA app was based on the design thinking method and user-centered approach, guided by older adults at every stage. Their input was pivotal, ensuring that the app’s features were tailored to their specific needs and preferences. Moreover, most existing apps in this domain predominantly cater to the younger segment of the older population, typically those aged 50 to 55 years [10,12,13,80-82]. This demographic focus leaves individuals aged ≥65 years underrepresented. The MIA app addresses this gap by concentrating research and design efforts on the age group of ≥65 years, thereby providing a more inclusive and representative technological solution for older adults.

Second, the MIA app incorporates behavior change theories such as the BCW [29], self-determination theory [33], and self-identification theory [30] to enhance user engagement.

Finally, the MIA app is engineered to dynamically adapt to the diverse capabilities and fluctuating motivations of its users [83,84]. It features an extensive repertoire of 195 distinct exercises encompassing various PA domains such as strength, coordination, balance, flexibility, and endurance, as well as incorporating activities pertinent to daily living. During the feasibility study, sending push notifications with personalized health messages was linked to increased user engagement. In addition, research indicates that users are more likely to interact with the app within 24 hours when push notifications are sent at midday on weekends [85-87]. Personalization is achieved by accounting for the inherently dynamic nature of motivation, which is known to vary not only from day to day but also within a single day [88]. To systematically capture these fluctuations, the app uses the technique of ecological momentary assessment [89]. This approach involves the intensive and recurrent collection of data on an individual’s behavior and motivation in a real-time environment [90-92]. Moreover, the app continuously refines its exercise recommendations based on user interactions and feedback. If an exercise is deemed excessively challenging, the algorithm records this, adjusting future workouts to better match the user’s current abilities and psychological state. This ensures that subsequent sessions are both enjoyable and attainable, fostering ongoing engagement and facilitating progressive improvement.

To ensure long-term retention and motivation, new videos and activities are regularly added to the app. Future updates to the MIA app will emphasize social features and gamification, including a buddy system for users to connect with exercise partners for mutual support, as well as earning badges, completing challenges, and participating in leaderboards for friendly competition. These elements, along with personalized workouts, progress tracking, content refreshers, and timely reminders, aim to enhance user engagement and sustained adherence.

### Strengths and Limitations

The results of this study should be interpreted in light of some limitations. First, the participant profile may not fully represent the broader older adult population. The individuals involved in this feasibility study were notably well educated and digitally literate, with nearly 60% (17/30, 57%) owning smartwatches. This contrasts with broader trends identified in a recent study [93], which found that only 25% of those aged ≥65 years use smartwatches or health apps, a number that declines to 16% among those aged >75 years. In our study, most participants already owned smartphones and were more acquainted with mobile technology. Future studies could explore the perceptions and willingness to adopt this mHealth app among older adults less familiar with such technology.

In addition, the participants who volunteered for this study might have been inherently more motivated and interested in PA than the general older adult population. This could limit the generalizability of the findings as their feedback might not reflect the perspectives of a broader, less technologically adept audience. Involving representatives of the older people demographic was very challenging because older adults are an extremely heterogeneous group with highly varied characteristics and needs who use, modify, and interact with technologies in rather diverse ways [94]. To mitigate the limitations of this study and enhance its generalizability, several strategies need to be implemented in the future. This involves targeting individuals who are less educated and less familiar with digital technology and those who do not own smart devices. Collaborating with general practitioners can help reach this more varied participant pool.

In addition, focus groups with older adults can sometimes result in conflicting opinions regarding an app’s design and features, which proves that a one-size-fits-all approach does not work for this population. Fortunately, the opinions of the older adults were well studied and were considered as much as possible during the iteration process after the feasibility study. To address the issue of conflicting opinions in focus groups, it would be beneficial to implement a more personalized approach in future studies [95,96]. This could include individual interviews that can capture a wider range of perspectives and experiences. This would allow researchers to explore deeper insights and accommodate each participant’s unique needs and preferences, moving beyond a one-size-fits-all solution.

To enhance both the reliability and validity of future studies, it is recommended to incorporate randomized controlled designs that include blinding procedures. This approach will help minimize bias by ensuring that assessors do not know which participants have received the intervention, thereby providing a more objective assessment of the outcomes [97,98]. In addition, conducting longitudinal assessments could yield a richer understanding of the intervention’s long-term attrition rates, further strengthening the study’s findings and their applicability across different settings and populations [99,100].

Finally, unrealistic expectations surrounding an mHealth app could pose a challenge. Users might expect immediate and significant improvements, failing to recognize that behavior change is typically a gradual process [24]. Therefore, it is crucial to underscore that the app is designed to be a supportive aid in the journey toward change, not a quick fix. To address this issue, managing expectations proactively is essential. Effective communication and educational initiatives are key to establishing a realistic understanding of what the app can and cannot do. By clearly outlining the app’s functionalities and limitations, users can be better prepared for their experience. This approach helps maintain sustained engagement and maximizes the app’s potential as a tool for positive transformation.

Despite these limitations, this study’s primary strength lay in its adoption of a mixed methods approach, which facilitated a comprehensive understanding of the app’s feasibility. The methodology combined surveys using validated tools, interviews, focus groups, and Microsoft Power BI analytics. This robust approach enabled us to construct a detailed profile of user behavior within the app. Through the systematic analysis of interaction data, we identified the features that garnered the most engagement and pinpointed areas for potential enhancement to improve user experience and retention. The insights derived from this extensive data analysis are fundamental in shaping the future development strategies of the app, ensuring that it aligns more closely with user needs and preferences. Furthermore, by using these diverse research techniques, we were able to gather rich data on participants’ experiences concerning the feasibility of the MIA app. This collaborative methodology proved especially beneficial, fostering meaningful dialogues between participants and researchers. These discussions yielded valuable perspectives on potential improvements to the mHealth app.

### Future Use and Implications in the Field

The MIA app is currently free and open source. However, to ensure its long-term viability, partnerships with health care insurers and other market players are being explored. A willingness-to-pay analysis [101], detailed in Multimedia Appendix 9, indicated that end users are willing to pay €4.50 to €7 (US $4.66 to US $7.25 at a conversion rate of €1=US $1.04) per month for access to the app. This willingness to pay suggests a perceived value of the app beyond its initial free access, which is further supported by user behavior. Specifically, after 6 months, 43% (13/30) of the participants continued using the app daily without being prompted.

Implementing MIA in real-world settings involves integrating it into health care systems, community programs, and support networks, positioning it as a preventive tool. Partnerships with health care providers and insurers can promote MIA’s role while community centers leverage its social features to encourage group activities.

As digital health technologies continue to evolve, the MIA app needs to take several opportunities into account to expand its applicability and feasibility. This offers a range of possibilities for the field.

Tailored user experience remains a very important subject to keep motivating older adults by providing relevant and achievable goals that cater to their individual fitness levels and health conditions [87,102,103]. Enhanced just-in-time interventions enabled by the app’s real-time data capabilities through ecological momentary assessment could revolutionize preventive health measures [104,105]. This merges with the potential for integration with wearable technologies, promising a more holistic approach to health monitoring that could improve predictive health interventions for older adults [106-108]. Moreover, integration with wearable devices can enhance the MIA app’s functionality by providing real-time, accurate data on PA levels and health metrics. This integration can promise a more holistic view of a user’s health and a more precise adjustment of their activity recommendations [50].

As social engagement appeared to be a critical component in maintaining motivation for PA among older adults, the MIA app could include more robust social networking features such as support groups, cooperative challenges, and shared fitness goals [30,109,110].

Another important evolution to consider is the evolution of advanced predictive analytics [111-113]. With advancements in AI and machine learning, the app could incorporate predictive analytics to forecast potential health risks based on user activity and health data. This feature could alert users and health care providers to potential health issues before they become severe, facilitating timely intervention.

In addition, the MIA app’s potential expansion to provide targeted support for informal caregivers, especially those managing care for partners with dementia, offers a pathway to significantly alleviate caregiver burden [114,115]. This feature could become increasingly vital during crisis situations such as pandemics, where the app’s adaptability could ensure continuous support for physical and mental health under restrictive conditions [116-119]. Furthermore, by enabling older adults to exercise independently, the app could empower health care professionals by reducing the frequency of in-person checkups, thereby optimizing health care resources [120].

Finally, a broader health integration more closely with health care systems allowing for a smoother exchange of information between the app and health care providers could be a great opportunity [121]. This integration could enable the development of personalized health care plans based on the app’s insights, enhancing the overall health care experience by keeping physicians informed and engaged in their patients’ lifestyle changes.

### Conclusions

This study highlights the potential in merging aging with technology to enhance quality of life and prevention through promotion of PA. Despite some limitations, the app received positive feedback for its usability and customer satisfaction. This underscores the value of involving users in the design process, adhering to a cocreation model that caters to their specific needs and counters age-related stereotypes in technology design. However, the insights gathered suggest a need for broader inclusivity in future studies, targeting less technologically savvy older adults to improve generalizability.

The analysis revealed strong engagement with specific app features (eg, workout videos) and highlighted areas for improvement, such as user interface and social connectivity enhancements. Longitudinal studies and ongoing iterations informed by user feedback will be crucial in refining these features. Managing expectations is also essential as technology adoption among older adults often requires recognizing that behavior change is gradual. Future directions include integrating the app into health care systems to tailor health plans more precisely to individual needs and expanding the app’s functionality using predictive analytics to pre-empt health issues. Ultimately, by continuing to evolve and adapt to user feedback and technological advancements, technology such as the MIA app can significantly contribute to promoting sustained, active, and healthy behaviors among older adults, demonstrating the profound impact of well-designed gerontechnology.

## Appendix

Multimedia Appendix 1 The full informed consent form used to secure participant consent for the study. The form is organized into the following sections: study overview, confidentiality and data privacy, participation and withdrawal details, contact information, consent declaration, and signature and date fields.

Multimedia Appendix 2 Inclusion and exclusion criteria for trial participation.

Multimedia Appendix 3 Think-aloud protocol.

Multimedia Appendix 4 Interview guide for the focus groups.

Multimedia Appendix 5 Complete acceptability results per category.

Multimedia Appendix 6 Visual representations of the Microsoft Power BI (Microsoft Corp) analytics.

Multimedia Appendix 7 Areas of improvement.

Multimedia Appendix 8 Strengths, weaknesses, opportunities, and threats matrix of the More In Action app according to the Nonadoption, Abandonment, Scale-up, Spread, and Sustainability components.

Multimedia Appendix 9 Willingness-to-pay analysis.

## Abbreviations

AI: artificial intelligence
BCW: Behavior Change Wheel
CONSORT: Consolidated Standards of Reporting Trials
CSAT: Customer Satisfaction Score
DHRQ: Digital Health Readiness Questionnaire
IPAQ-SF: International Physical Activity Questionnaire–Short Form
mHealth: mobile health
MIA: More In Action
NASSS: Nonadoption, Abandonment, Scale-up, Spread, and Sustainability
NPS: Net Promoter Score
PA: physical activity
PACES: Physical Activity Enjoyment Scale
SUS: System Usability Scale
