# Supplementary material for: Exploring the Feasibility of a 5-Week mHealth Intervention to Enhance Physical Activity and an Active, Healthy Lifestyle in Community-Dwelling Older Adults: Mixed Methods Study
Source: JMIR Aging. 2025 Jan 27;8:e63348. doi: 10.2196/63348 (PMC11811674; doi:10.2196/63348)
Supplement: Multimedia Appendix 1 [file aging_v8i1e63348_app1.docx]

# Appendix 1 Informed consent form

## PART 1: INFORMATION LETTER

**Participating in a scientific study, what does that mean to you?**

*Study Title: The Importance of an Active Lifestyle: Promoting physical activity among older adults through the use of a mobile application.*

Dear Sir/Madam,

With this information letter we would like to ask you if you would like to participate in a scientific study. Participation is voluntary. In this letter you can read what kind of study it is, what it means for you, and what the advantages and disadvantages are. It's a lot of information. Would you like to read through the information and decide if you want to participate? If you would like to participate, please fill out the form in Appendix B.

**Ask your questions**

You can make the decision to participate based on the information you will find in this information letter. In addition, we recommend that you do this:

- Ask questions to the researcher who gives you this information.

- Talk to your partner, family or friends about this study.

**What information can you find in this information letter?**

You will find this information in this letter:

- What does this study involve?
- Why am I being asked to participate?
- Do I have to participate in a study?
- What will happen during the study?
- Will I benefit from the study?
- What are the potential risks and inconveniences of participating in the study?
- What data is collected and how do we handle your data?
- Can I end my participation in the study early?
- What do we expect from you if you participate in this study?
- Do I get paid? Or will my participation in the study entail additional costs for me?
- Am I specially insured for this study?
- When does the study end?
- Who reviewed and approved the documents relating to the study?

You will find the following attachments to this information letter:

A: contact details of PXL University of Applied Sciences

B: consent form

1. **Why are we doing this study?**

PXL University of Applied Sciences has set up this study to investigate how we can motivate people from the age of 65 to exercise more by means of a mobile exercise program. This exercise program, called MIA, will work on both a smartphone and a tablet.

1. **Why am I being asked to participate?**

You have indicated that you are interested in participating in this study. In addition, you meet the inclusion criteria for this study, which are:

- ≥65 years of age at the start of study (i.e., born on or before October 9, 1958)
- have a good understanding of the Dutch language and actively speak it
- Independent living (in a detached house or serviced flat/assisted living facility)
- do not suffer from one or more of the following (chronic) conditions:
  - type 2 diabetes (i.e., both insulin-dependent and non-insulin-dependent type 2 diabetes that involves taking oral medications or using injections)
  - Multiple Sclerosis (MS)
  - Parkinson's disease
  - cardiovascular disease (<5 years ago) (e.g. stroke, myocardial infarction, bypass/bridging surgery, stenting)
- do not suffer from a neurological disorder that was recently diagnosed (<1 year ago)
- have not undergone surgery in the recent past (<6 months ago)

1. **Do I have to participate in a study?**

Participation in a study is voluntary and should never be done under pressure. This means that you have the right not to participate in the study. You may also withdraw from the study at any time without having to give a reason, even if you have previously agreed to participate. Your decision will not affect your relationship with the investigator.

1. **What will happen during the study?**

You will visit PXL University of Applied Sciences twice (Campus Healthcare, Guffenslaan 39, 3500 Hasselt).

The first session will take about 1.5 hours. The following aspects will be questioned:

- General data (gender, marital status, highest degree obtained);
- Your level of physical activity during the past 7 days;
- Your enjoyment of physical activity;
- Your use of technology;
- Your first impressions about the MIA exercise program.

During this first session, the MIA app will be installed on your smartphone or tablet and you will receive an explanation of how it works.

You will then work independently with the MIA exercise program for 5 weeks.

At the end of this trial period, you will visit PXL University of Applied Sciences again. The second session will take about 2 hours to complete. The following aspects will be questioned:

- Your enjoyment of physical activity;
- Your assessment of the MIA app (both through *individual* questions and through a *group discussion* (regarding your first impression, technical aspects, specific aspects about the use and user-friendliness of the app).

1. **Will I benefit from the study?**

Your participation in this study can contribute to a better understanding of how people from the age of 65 can be stimulated to exercise more via a mobile application.

It is possible that you also experience the benefits nl. dat you enjoy using the exercise program and that you feel that your physical condition improves. By feeling fitter, you may feel that you can perform daily activities such as climbing stairs, household activities and garden work better.

1. **What are the potential risks and inconveniences of participating in the study?**

The exercise program is designed in such a way that exercises are offered in different levels of difficulty (beginner – advanced – expert). All exercises will also be provided with a video with spoken instruction so that it is clear how (and with what necessary materials) the exercises can be performed safely.

However, it can never be ruled out that you fall or suffer a minor injury (e.g. sprain, overuse). Experiencing mild fatigue or muscle pain can also occur as a result of following this exercise program for weeks.

However, scientific literature has shown that the health benefits of exercise (positive impact on endurance, muscle strength, body weight, cholesterol, reduced risk of cardiovascular disease, etc.) are so great that participation in the exercise program can be considered beneficial for physical and mental health. Moreover, to date, there is no evidence whatsoever that exercise could have any adverse effect on your health.

Secondly, you should be aware that data is being recorded about you. This data can only be viewed by the researchers. You can find more information about this below (point 7).

1. **What data is collected and how do we handle your data?**

In this study, the following data will be collected:

- General data (gender, marital status, highest degree obtained);
- Your level of physical activity during the past 7 days;
- Your enjoyment of physical activity;
- Your use of technology;
- Your first impressions about the MIA exercise program.
- Your assessment of the MIA app (both through *individual* questions and through a *group discussion* (regarding your first impression, technical aspects, specific aspects about the use and user-friendliness of the app).

The researcher is bound by professional secrecy when collecting and processing your data. This means that he/she will never reveal your identity, not even in a scientific publication or a lecture, and that he/she will encrypt your data (i.e. replace your identity with an identifier in the study). As a result, the investigator, and the study staff under the responsibility of the investigator, will be the only ones who will be able to link your identity to the data recorded during the study.

There will be 4 simultaneous ways to securely store all data associated with this research project:

1. Each researcher has the use of a personal database on the laptop that can only be accessed via his/her unique staff number and personal password (= "Cloud").

2. "Microsoft Authenticator" is used as a "double security step". This means that, before files on the laptop can be opened, a unique code must first be entered that can only be consulted via the Microsoft Authenticator app on the researcher's personal smartphone.

3. The password on each researcher's laptop must be changed every 6 months.

4. All files containing data relating to the research project will be protected by a password, which will only be known to the researchers involved.

In accordance with the GDPR legislation, the data is kept for a maximum of 20 years. You also have the right to view the data yourself.

1. **Can I end my participation in the study early?**

You participate in this study voluntarily and you have the right to withdraw your consent for any reason. You don't have to give a reason for this. If you withdraw your consent, the data collected up to the time of discontinuation will be retained in order to guarantee the validity of the study. No new data will be recorded.

1. **What do we expect from you if you participate in this study?**

If you participate in this study, we ask you to: 1) Fully cooperate for the proper conduct of the study, and 2) Not to conceal information about your health status or the symptoms you are experiencing.

1. **Do I get paid? Or will my participation in the study entail additional costs for me?**

You will not be paid to participate in this study. Participation is voluntary and is done on the one hand to get acquainted with the possibilities that the exercise program can offer you and on the other hand to help science. With the exception of your travel costs (to come to Hasselt twice for the initial and final evaluation), there are no costs for you to participate in this study.

1. **Am I specially insured for this study?**

The sponsor is liable, even if there is no fault, for the damage that you as a participant - or in the event of death your beneficiaries - incur and that is directly or indirectly attributable to participation in this study. The client has taken out an insurance contract for this purpose.

1. **When does the study end?**

The study will stop after 6 weeks when the questions and interviews of the second session (final session) have been conducted. Furthermore, you have the right to stop at any time without giving a reason. Participation is completely voluntary.

1. **Who reviewed and approved the documents relating to the study?**

The study documents were reviewed by an independent Belgian Ethics Committee, namely the Ethics Committee of Hasselt University.

Ethics committees have the task of protecting the people who participate in a study. The competent health authorities will ensure that the study is carried out in accordance with the applicable legislation. You should not take their approval as an incentive to participate in the study.**APPENDIX A: CONTACT DETAILS OF PXL UNIVERSITY OF APPLIED SCIENCES**

This research is carried out from:

PXL University of Applied Sciences

Expertise Centre for Healthcare Innovation and Expertise Centre Smart-ICT

Guffenslaan 39

3500 Hasselt

Belgium

Coordinating Principal Investigator Contact Information:

Kim Daniels

Local Principal Investigator Contact Details:

dr. Nastasia Marinus

dr. Ryanne Lemmens

**APPENDIX B: CONSENT FORM**

*Study Title: The Importance of an Active Lifestyle: Promoting physical activity among older adults through the use of a mobile application.*

# Informed consent

# **Participant**

- I declare that I have been informed about the purpose of the study, its duration and consequences, possible risks and inconveniences and what is expected of me, and that I have understood all this. My rights as a participant in a study have been explained to me and I have understood them.
- I have had enough time to think about it and talk about it with a confidant (e.g. friends, family, attending physician, etc.).
- I have had the opportunity to ask all the questions that came to my mind and I have received a satisfactory answer.
- I understand that I will participate in this study voluntarily and without being forced to do so and that I can stop my participation in the study at any time.
- I understand that data about me will be collected and treated confidentially.
- I understand that the client has taken out insurance in case I would suffer damage in connection with my participation in this study.
- I understand that I do not incur any costs (except for travel expenses) when participating in this study.
- I agree that I will not participate in another study at the same time without informing the investigator or study staff, and that they may refuse to participate for justified reasons.
- I understand that I must cooperate and follow the instructions of the investigator and of the study staff around the study.
- I understand that my participation in the study may be terminated without my consent if I require a different treatment, do not follow the study schedule, have an injury related to the study, or for any other justifiable reason.
- I confirm that all the information I have given about my medical history is correct. I understand that it may cause me harm if I fail to inform or point out possible exclusion criteria to the investigator.

I agree to participate in the study, and I have received a signed and dated copy of all pages of this document.

Name and surname of the participant:

Date:

Signature of the participant:

Name and surname of the researcher:

Date:

Signature of the investigator:
