# Supplementary material for: Exploring the Feasibility of a 5-Week mHealth Intervention to Enhance Physical Activity and an Active, Healthy Lifestyle in Community-Dwelling Older Adults: Mixed Methods Study
Source: JMIR Aging. 2025 Jan 27;8:e63348. doi: 10.2196/63348 (PMC11811674; doi:10.2196/63348)
Supplement: Multimedia Appendix 2 [file aging_v8i1e63348_app2.docx]

# Appendix 2: Inclusion and exclusion criteria for trial participation

| Inclusion criteria | Exclusion criteria |
| --- | --- |
| Participants are 65 years of older | Current neurological disorder such as Parkinson’s disease, multiple sclerosis, cerebrovascular accident, … |
| Participants are competent to give informed consent | Current cardiovascular disorder such as stroke, acute myocardial infarct, coronary artery bypass grafting, percutaneous coronary intervention less than 5 years ago |
| Participants can actively participate in the study | Current respiratory disorder, such as chronic obstructive pulmonary disease, pneumonia, pulmonary fibrosis, asthma, … |
| Participants are community-dwelling (living independent at home or in a service apartment) | Current severe metabolic disorder, such as diabetes type 1 and 2, severe osteoporosis, … |
| Without a severe illness | Current severe cognitive disorders, such as Alzheimer’s disease, vascular dementia, Lewy Body dementia, frontotemporal dementia. |
| Dutch language proficiency as native speaker |  |
