# Supplementary material for: Exploring the Feasibility of a 5-Week mHealth Intervention to Enhance Physical Activity and an Active, Healthy Lifestyle in Community-Dwelling Older Adults: Mixed Methods Study
Source: JMIR Aging. 2025 Jan 27;8:e63348. doi: 10.2196/63348 (PMC11811674; doi:10.2196/63348)
Supplement: Multimedia Appendix 3 [file aging_v8i1e63348_app3.docx]

# Appendix 3: Flow Think – aloud protocol

GOAL: To capture the first findings of participants about the MIA exercise

*Info for the participant*

I'm going to ask you to think out loud as you do the tasks. Let me explain what I mean by "thinking out loud." It means that I would like you to tell me everything that is on your mind as you perform each task. When I say tell me everything, I really mean every thought you have from the moment you get the task to when you finish the task. Please don't worry about how you say things or how you convey your thoughts to me. What I really want is for me to constantly hear your thoughts as you perform the task, I imagine you may find it uncomfortable to think out loud but try to imagine that you are alone in the room. If it gets quiet for too long, I say "keep talking" to remind you to think out loud. Please note, this research is very exploratory. It is not my intention to evaluate your thinking or explanations as you speak. The goal of the study is to learn more about the thoughts as you – and other people – perform each task. Your findings will be treated confidentially. The recording will only be used for study purposes.

| **TASK** | **Participant's findings** |
| --- | --- |
| Open the exercise app MIA |  |
| View the welcome screen |  |
| Navigate to my profile |  |
| Complete your profile by filling out the questionnaire |  |
| Navigate to the home screen |  |
| Take a look at the home screen and its components |  |

| **TASK** | **Participant's findings** |
| --- | --- |
| Navigate to "Ask a Question" (Help Desk) |  |
| Check out the helpdesk |  |
| Ask a test question in the helpdesk |  |
| Take a look at the visual aspect of MIA in its entirety |  |

| **TASK** | **Participant's findings** |
| --- | --- |
| Use the smileys to indicate how you feel today |  |
| Click on any suggested workout on the home page |  |
| Navigate to the workouts page |  |
| Check out the workouts page and its components |  |
| Click on any workout and take a look at it |  |
| Navigate to the calendar |  |
| View the calendar page |  |
| Click on any calendar item |  |
| Navigate to learning |  |
| **TASK** | **Participant's findings** |
| Check out the learning page |  |
| Click on any theme from the learning page and choose an item, take a look at it |  |
| Navigate to the diary |  |
| Add an exercise activity from last week to the diary |  |
| Navigate to overview |  |
| View the overview page |  |
| Navigate to the manual |  |
| View the guide |  |

Do you have any other comments and possible solutions/ideas for this?

........................................................................................................................................................................................................................................................................................................................

........................................................................................................................................................................................................................................................................................................................

........................................................................................................................................................................................................................................................................................................................

Do you have any tips for MIA?

........................................................................................................................................................................................................................................................................................................................
