# Supplementary material for: Exploring the Feasibility of a 5-Week mHealth Intervention to Enhance Physical Activity and an Active, Healthy Lifestyle in Community-Dwelling Older Adults: Mixed Methods Study
Source: JMIR Aging. 2025 Jan 27;8:e63348. doi: 10.2196/63348 (PMC11811674; doi:10.2196/63348)
Supplement: Multimedia Appendix 4 [file aging_v8i1e63348_app4.docx]

# Appendix 4: Interview guide focus groups

|  | **Main question** | **Asking questions** |
| --- | --- | --- |
| **PERFORMANCE** | | |
| 10.00 – 10.10 | - Introduction - You have all already filled in the questionnaires in which you could already give your opinion about the app. Now we would like to talk to you to see in more detail what you thought of MIA. - What you think could be improved or is already good and what may still be missing. - The goal for us is to make the MIA app as good as possible. And that's why we think it's so nice and important that you were the first to try out MIA and share your experiences with us! - What are we going to do? We will soon go through all the parts of the app together and ask some questions about this. - You can give your opinion on this. And there is no wrong or right answer here. We think it's important to find out what your opinion is. - It may well be that you don't agree with each other and that's totally fine. - Furthermore, you can also be very critical: We can handle that and only in this way can we make MIA better. - We will also make an audio recording. This one is purely for us to listen to afterwards because it is difficult to write everything right away. No names are recorded, so all is anonymous. - It will last until about 12 o’clock. - Presentation of researchers with the name | |
| **FIRST IMPRESSIONS** | | |
| 10.10 – 10.20 | - What is your first impression of the app as you used it? - "Which parts of the app did you use the most?" | - Did you succeed in using the app? |
| **HOME** | | |
| 10.20– 10.30 | - Are there things you missed on the homepage? - What did you think of setting an exercise goal? | - - - What do you think of the range of information on the homepage?     - What kind of information are you missing from this page? |
| **WORK-OUTS** | | |
| 10.30 – 10.40 am | - - What did you think of the way the exercise videos were presented and explained?   - You could follow along with the exercises well in terms of pace, explanation, speed, clarity, explanation   - Did you have to stop video at the instruction?   - How did you solve it when an exercise didn't work out so well?   - How was it easy to practice with equipment? |  |
| **AGENDA** | | |
| 10:40 a.m. – 10:45 a.m. | - The agenda is there because of the social connection with others. Has the calendar contributed to this or how could it be otherwise? - What could we improve about the calendar? | - Have you used it - Have now gone to an activity. |
| **LEARN** | | |
| 10:45 a.m. – 11:55 a.m. | - - Did you learn more about health and the importance of exercise through the learning item?   - Have you changed your behavior because of the new things you have learned? | - - What else could we improve/add? |
| **DIARY** | | |
| 10:55 a.m. – 11:05 a.m. | - - Did you keep track of your activities in the past? Have you done it now and why did you like it? | - What do you think of the clarity of the diary? - Did that work out well? |
| **OVERVIEW PAGE** | | |
| 11:05 a.m. – 11:10 a.m. | - - What did you think of the overview page?   - What would you advise us to change about this? | - How do you feel about us tracking your progress while using the app? |
| **PROFILE** | | |
| 11.10 am – 11.15 am | - - We asked a number of questions at the start to make the app more tailored to your personal preferences. Do you feel that you have succeeded? And in what way or not? | - - What would you recommend us to change about the profile?   - What did you like about the profile?   - What did you think of setting the exercise frequency (number of days/hours) in your profile? |
| **ASK A QUESTION** | | |
| 11.15 – 11.20 | - Is it a useful feature and why or why not? | - - What was your experience with this? |
| **LAYOUT** | | |
| 11.20 – 11.25 | - What did you think of the readability of the texts? - What did you think of the layout of the app? - What would you recommend us change about the layout of the app? | - - What did you like about the app's layout?   - What did you dislike about the app's layout?   - How did you like the colors? |
| **ADDED VALUE OF MIA** | | |
| 11:25 a.m. – 11:35 a.m. | - What is the added value of MIA compared to your motivation to exercise? - Which part of the MIA app has encouraged you to move more? - How did you fit the app into your daily life? Has it become routine? - How does the mia app compare to other initiatives to get more exercise? Is it better or not? | - Do you do certain activities more in your daily life now?' - 'Do you think the app can contribute to a better execution of these activities? |
| **FUTURE USE** | | |
| 11:35 a.m. – 11:45 | - Why would you or wouldn't you want to use the app in the future? |  |
| **GENERAL** | | |
| 11:45 a.m. – 11:45 a.m. | - What kind of technical issues did you experience while using the app? - How could we make the app even better? - What did you find superfluous about the app? - What else should definitely be added to the app for you? | - What did you do in case of a technical problem with the app? |
| **FINALLY** | | |
| 11:55 a.m. – 12:00 p.m. | - Is there anything else we didn't ask that you would like to tell us? |  |
