# Supplementary figures and images for: Exploring the Feasibility of a 5-Week mHealth Intervention to Enhance Physical Activity and an Active, Healthy Lifestyle in Community-Dwelling Older Adults: Mixed Methods Study
Source: JMIR Aging. 2025 Jan 27;8:e63348. doi: 10.2196/63348 (PMC11811674; doi:10.2196/63348)

# Appendix 5: Complete results of the acceptability per category


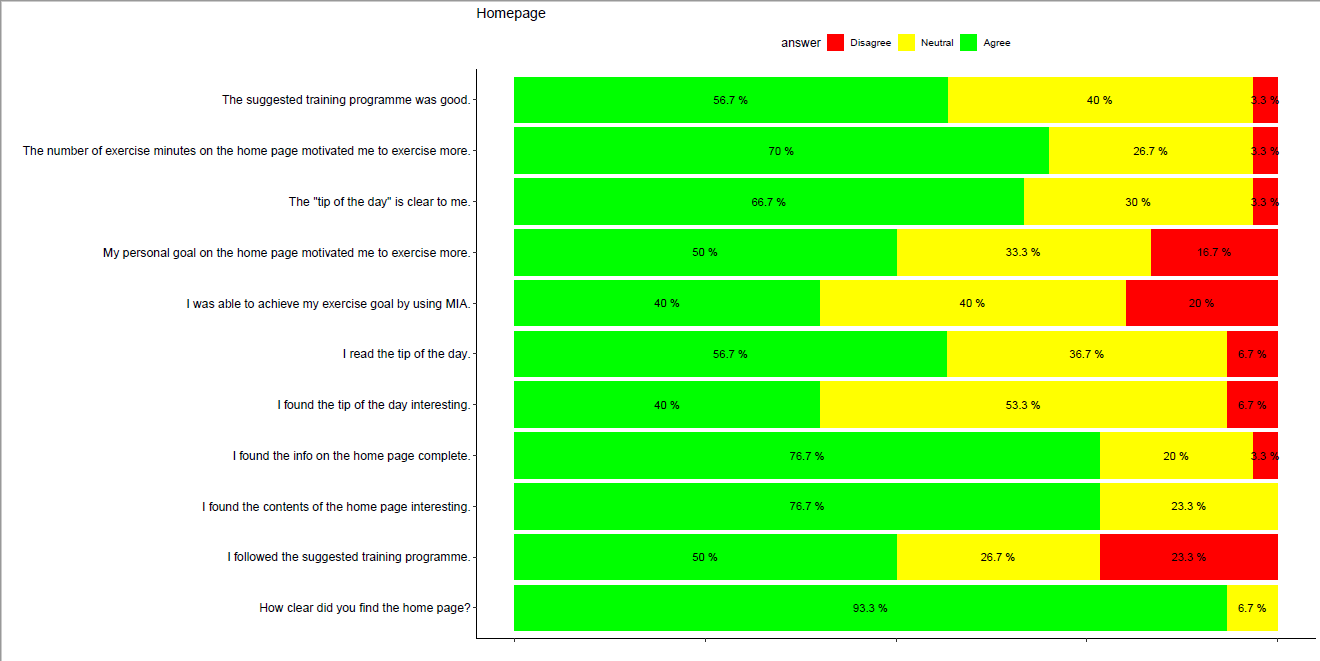


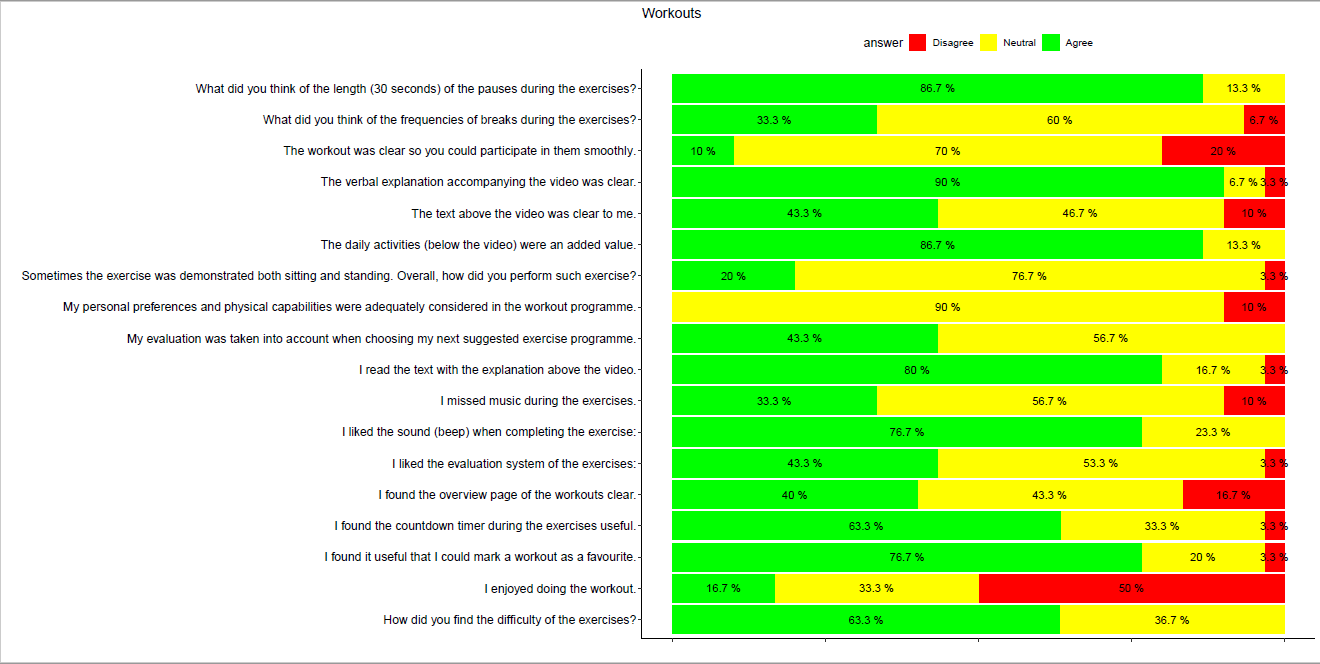


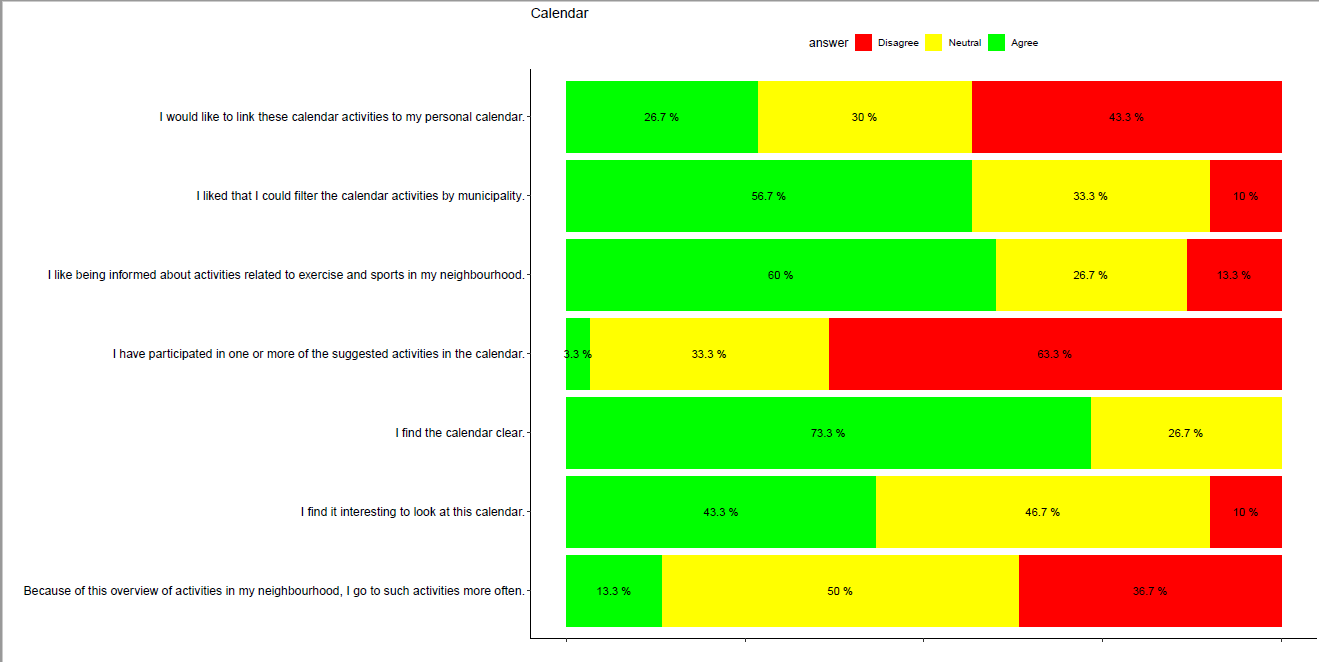


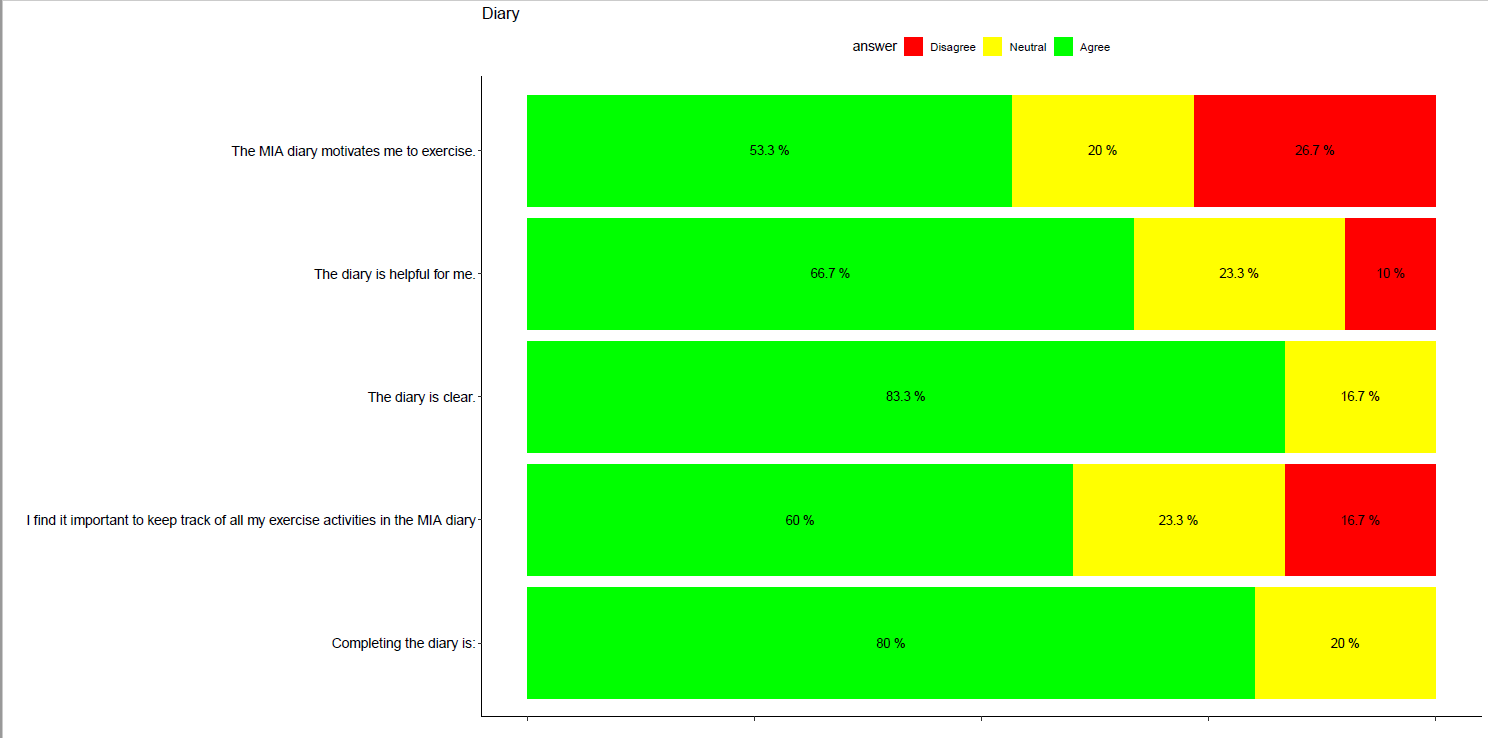


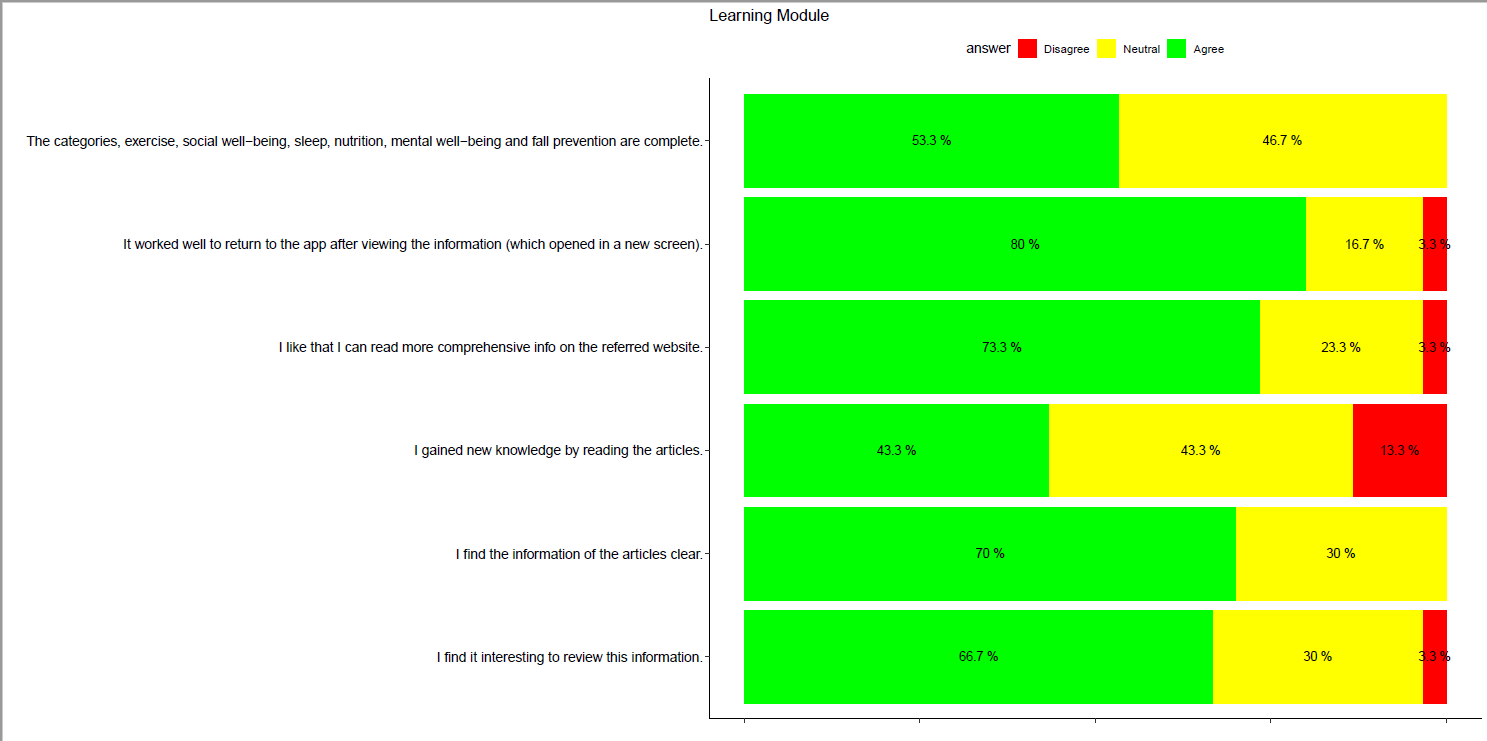


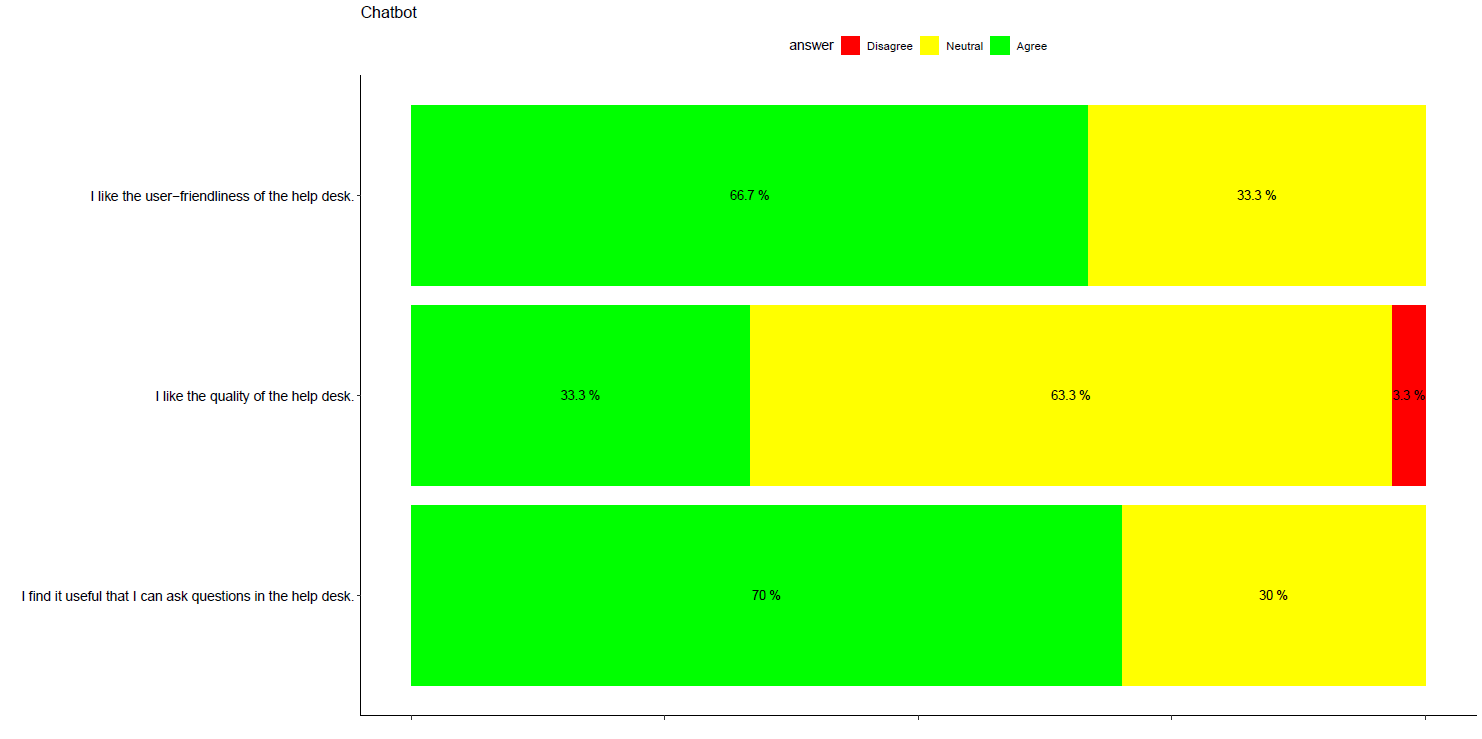

Supplement: Multimedia Appendix 5 [file aging_v8i1e63348_app5.docx]
