# Supplementary material for: Exploring the Feasibility of a 5-Week mHealth Intervention to Enhance Physical Activity and an Active, Healthy Lifestyle in Community-Dwelling Older Adults: Mixed Methods Study
Source: JMIR Aging. 2025 Jan 27;8:e63348. doi: 10.2196/63348 (PMC11811674; doi:10.2196/63348)
Supplement: Multimedia Appendix 6 [file aging_v8i1e63348_app6.docx]

# Appendix 6: Visual representations of the Power BI analytics

**
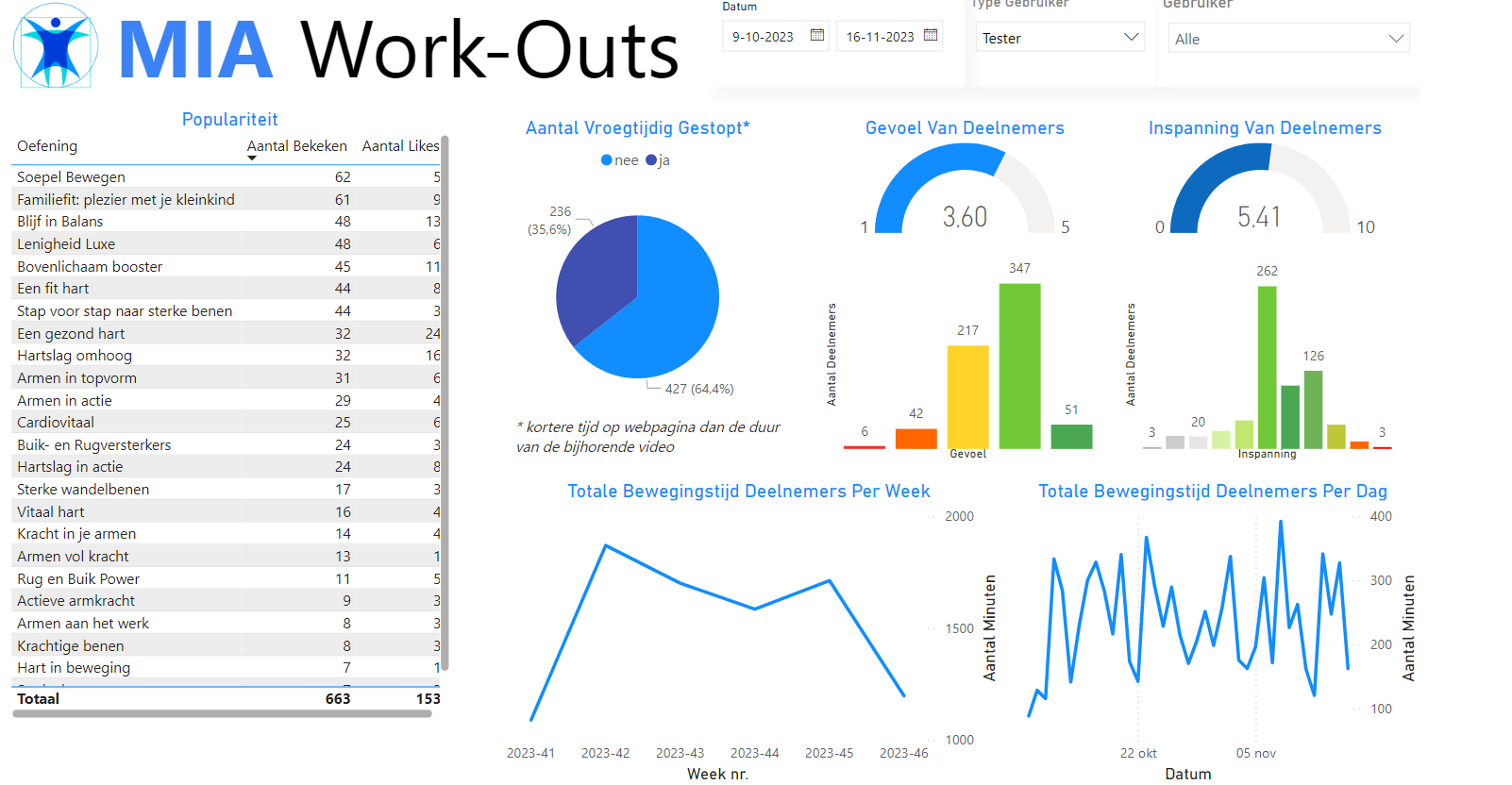
**

Power Bi on the work-out videos

**
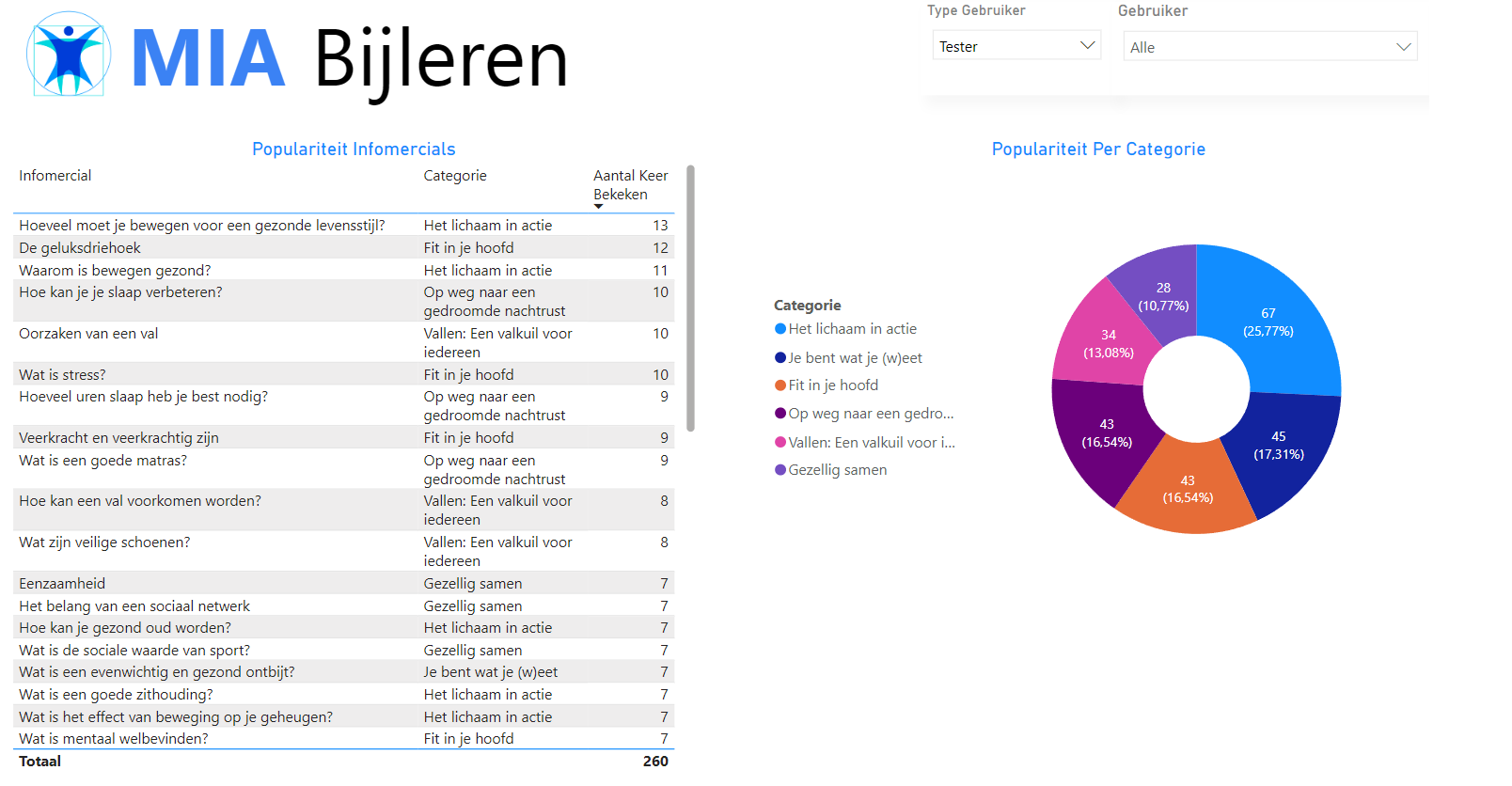
**

Power Bi on the leaning module

**
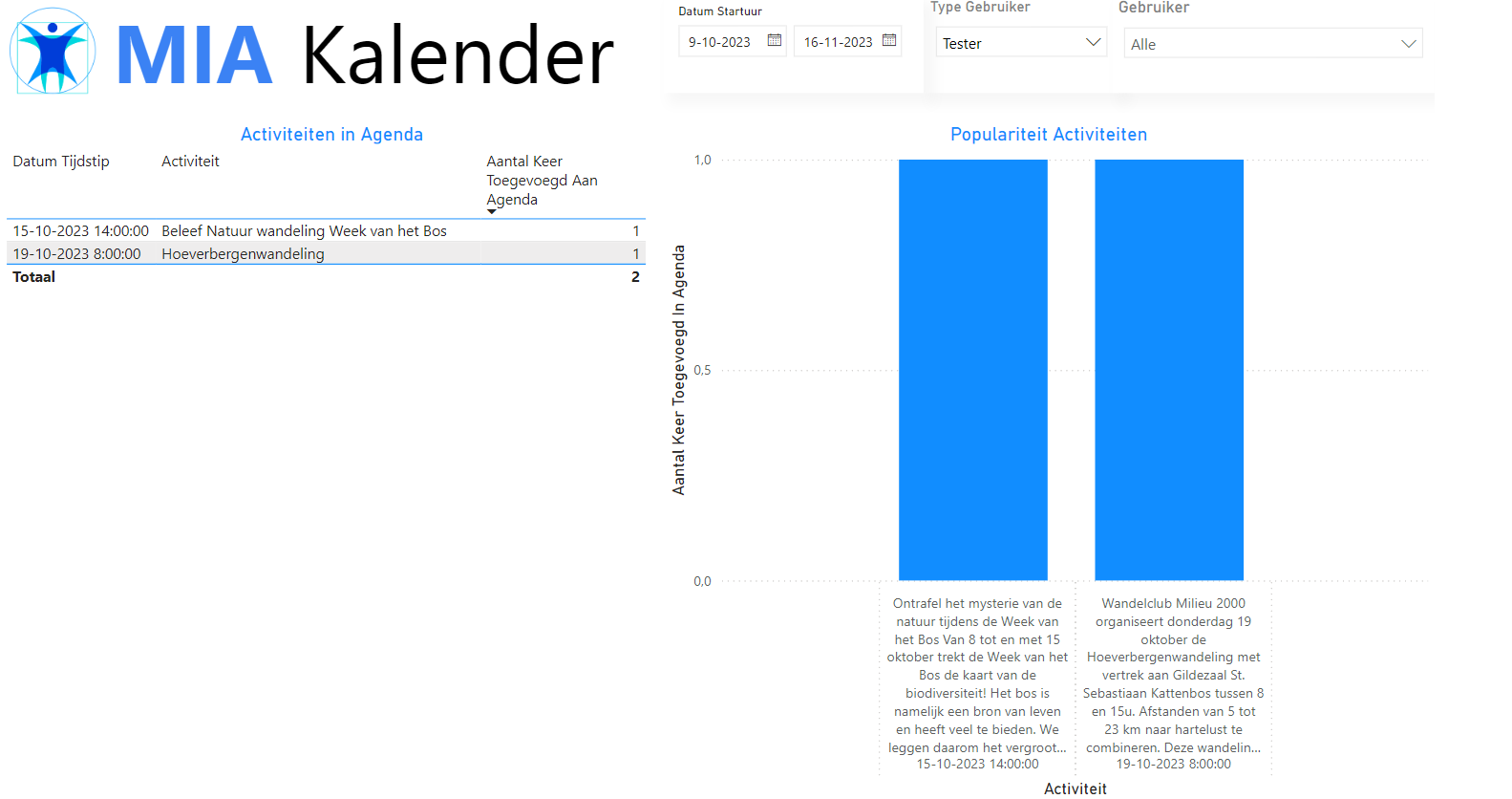
**

Power Bi on the community calendar

**
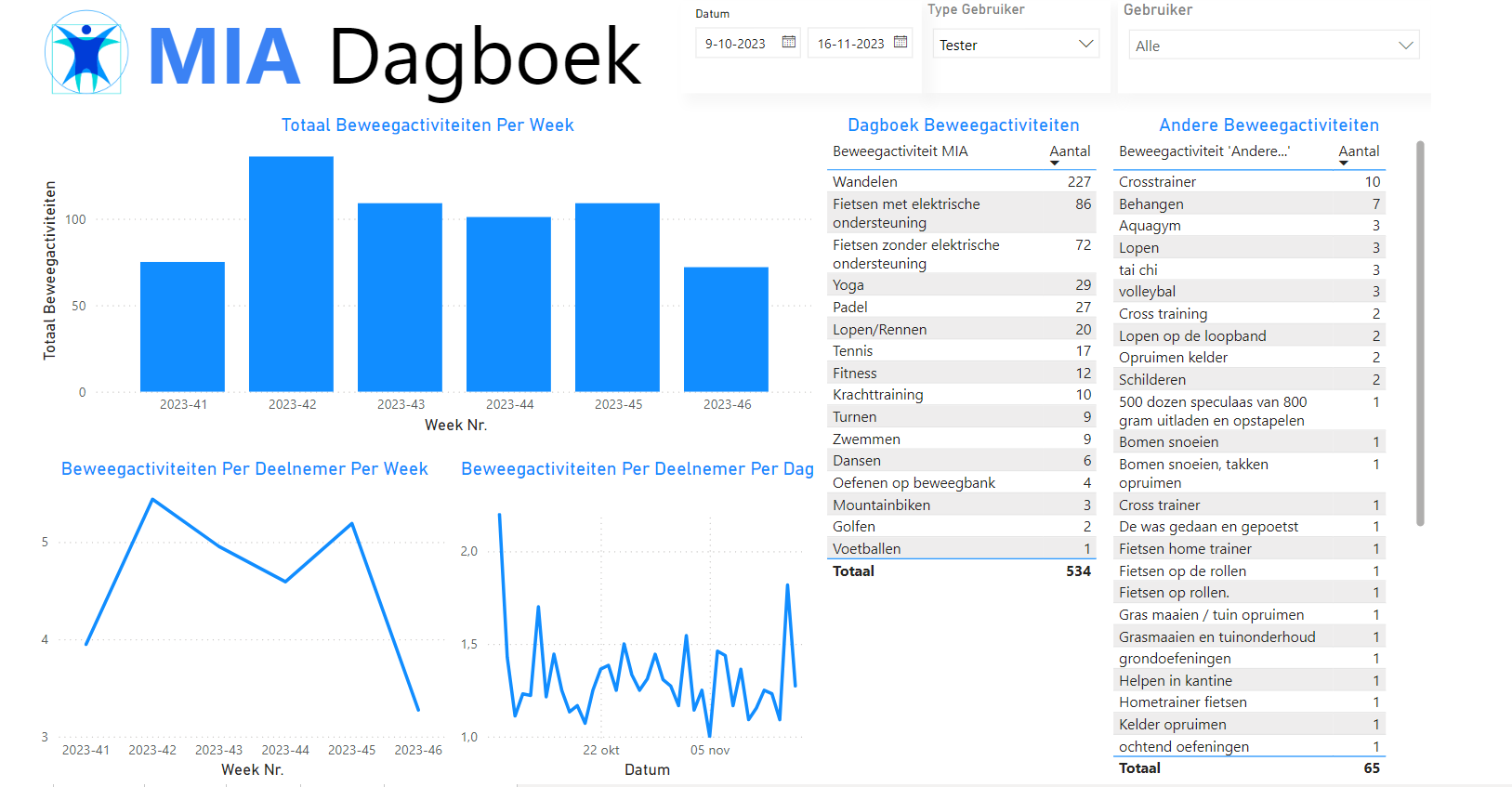
**

Power Bi on the manual diary

**
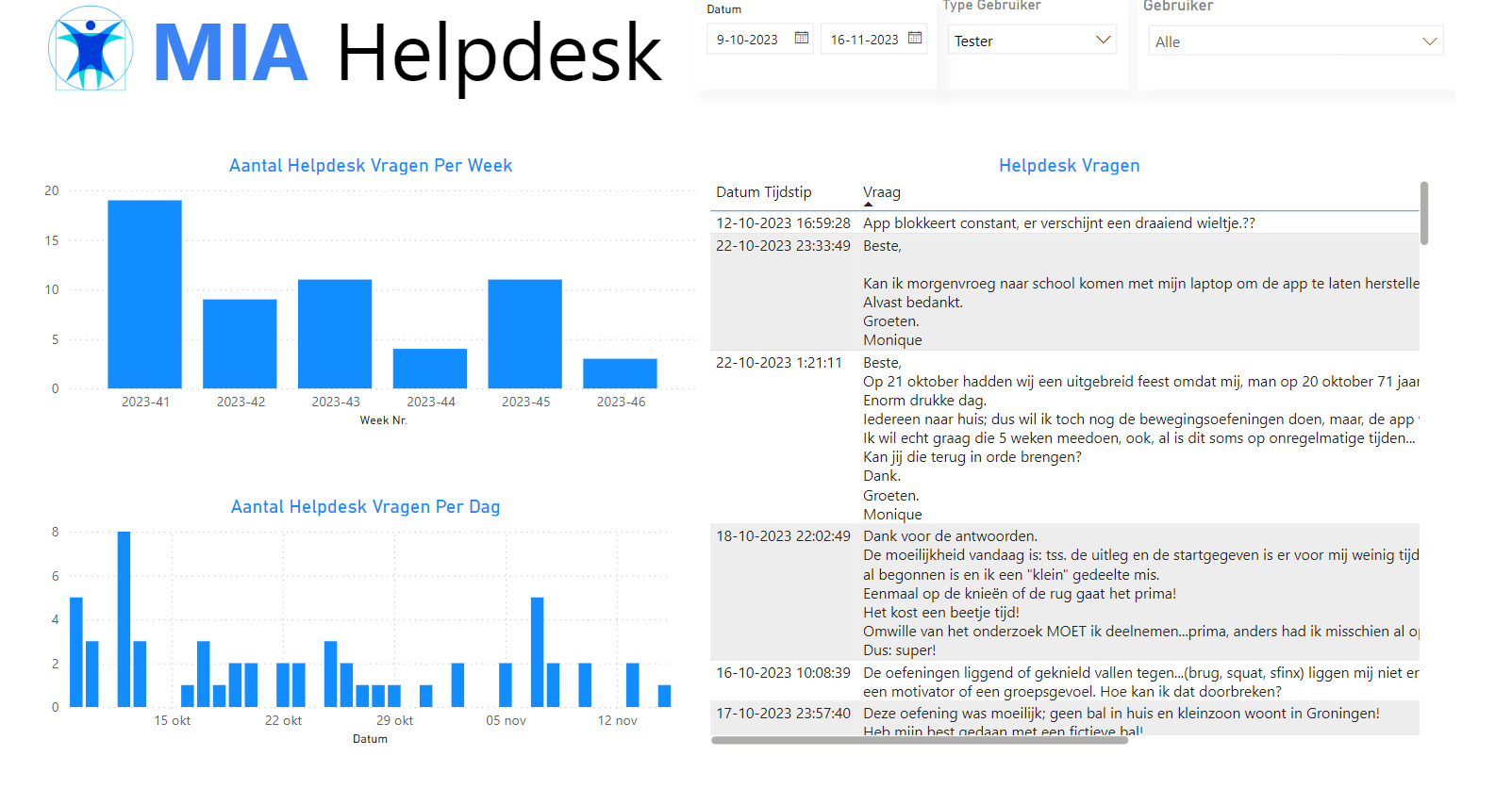
**

Power Bi on the chatbot
