# Supplementary material for: Exploring the Feasibility of a 5-Week mHealth Intervention to Enhance Physical Activity and an Active, Healthy Lifestyle in Community-Dwelling Older Adults: Mixed Methods Study
Source: JMIR Aging. 2025 Jan 27;8:e63348. doi: 10.2196/63348 (PMC11811674; doi:10.2196/63348)
Supplement: Multimedia Appendix 7 [file aging_v8i1e63348_app7.docx]

# Appendix 7: Areas of improvement

| MIA General | | | | | | | |
| --- | --- | --- | --- | --- | --- | --- | --- |
| Problem | **Problem in detail** | **Possible solution** | **Execute** | **Complexity** | **Priority** | **Responsbility** |  |
| Spelling | There were spelling mistakes in it | Check everything again | Yes | Low | High | Researcher |  |
| Accessibility | Too small to read on a smartphone | Make it smartphone friendly | No | Low | Low | Engineer |  |
| Calendar | | | | | | | |
| Range of activities | Activities far from your place of residence | Offer more activities | Yes | Low | Low | Researcher |  |
|  | Not all activities in the region are in the list | Offer more activities | Yes | Low | Low | Researcher |  |
|  |  | Offer the possibility to submit / upload activities themselves | No | High | Low | Researcher |  |
|  | It needs to stay updated | Investigate automation | Yes | High | Low | Researcher |  |
|  | Price of activity was not always mentioned | Mention price in text | Yes | Low | Low | Researcher |  |
|  | Walking distance was not always mentioned | Mention distance | Yes | Low | Low | Researcher |  |
| Clarity of calender | Not clear that you can click for more info | Mention 'click for more info', in tutorial | Yes | High | Low | Researcher |  |
|  | Would be useful if you could enter your own location and then see all the activities around it | Don't just filter by municipality, but enter municipality and sort by distance | Yes | High | Low | Engineer |  |
| Social aspect | No possibility to meet up with others in the app to go together | Investigate what is possible to connect or buddy | No | High | Low | Engineer |  |
| WORKOUTS | | | | | | | |
| Unclear what material they need | No Extra Info | Make a list of materials in the text above the workout | Yes | Low | High | Engineer |  |
|  |  | Mention at the beginning of the video | No | High | Low | Researcher |  |
| Workout video's | Pumping exercise and some others too heavy for beginner | Adjusting workouts and pumping exercise not for beginners, instructional video | Yes | High | High | Researcher |  |
|  | Tempo exercises too high for some | It should be clearly indicated that people can adjust the WO (pace, difficulty, skip it) according to their own abilities | No | Low | High | Researcher |  |
|  |  | Indicate in the instruction video on the app that you can perform the exercise at your own pace | Yes | High | High | Researcher |  |
|  |  | Add to daily tips | Yes | Low | High | Researcher |  |
|  |  | Voice over let it indicate that man is at your own pace | No | High | Low | Researcher |  |
|  | Pause between exercises too long | - indicate what they can do during the break, e.g. go out or have a drink - via voice over | No | High | Low | Researcher |  |
|  |  | - indicate what they can do during the break, e.g. go out or have a drink- via instructional video | Yes | Low | High | Researcher |  |
|  |  | - indicate what they can do during the break, e.g. go out or have a drink- via text on the break screen | Yes | Low | Low | Researcher |  |
|  |  | How to make pause customizable in profile | No | High | Low | Engineer |  |
|  | Break too short | - indicate somewhere that you can also manually pause to extend pause, e.g. instruction video | Yes | Low | High | Researcher |  |
|  |  | - indicate somewhere that you can also manually pause to extend pause - via text pause screen | No | High | High | Researcher |  |
|  |  | How to make pause customizable in profile | No | High | Low | Engineer |  |
|  | Length workouts | Opinions were divided, too short, too long, good | No | Low | Low | Researcher |  |
|  | No overview of how long the workout will take or how many exercises are still to come | View progress per workout in a timeline | Yes | High | High | Researcher |  |
|  | Too little variation | Offering more different WO | Yes | Low | High | Researcher |  |
|  |  | Incontinence exercises were missing --> add | No | High | Low | Researcher |  |
|  |  | Also make a workout with a combination of strength, endurance, balance, etc., so not just 1 domain | Yes | Low | High | Researcher |  |
|  | Floor exercises difficult | Do not offer to beginners | Yes | Low | High | Researcher |  |
|  |  | Filter by No Floor Exercises | Yes | Low | High | Engineer |  |
|  |  | Giving more time to switch from standing to floor exercise | Yes | Low | High | Researcher |  |
|  |  | indicate that they can skip it if it is difficult on the ground | Yes | Low | High | Researcher |  |
|  | Voiceover less pleasant/too much chatter | Less voice in new workouts | No | Low | Low | Researcher |  |
|  |  | A different voice for new workouts | No | High | Low | Researcher |  |
|  | Voiceover description exercise on the late side |  | No | High | Low | Researcher |  |
|  | Music was missed and not | Want to offer music optionally? So all workouts 2x on it, 1x with and 1x without music and indicate in profile what they want and filter on that | No | High | Low | Researcher |  |
|  |  | Separate Spotify list | Yes | Low | High | Researcher |  |
|  | Difficulty level | Workouts too hard or not hard enough | No | Low | High | Researcher |  |
|  |  | indicate that they can perform an exercise more powerfully or make it easier, e.g. in instruction movie | Yes | Low | High | Researcher |  |
|  | Correct execution is not checked | Possibly set up cooperation with students Education to give the opportunity to exercise under supervision once in a while | No | Low | Low | Researcher |  |
|  |  | Virtual Coach | No | High | Low | Researcher |  |
|  |  | Kine as a coach | No | High | Low | Researcher |  |
|  | Build-up workouts not clear in terms of warming up and cooling down | Create an intermediate screen with warming up, core and cooling down | Yes | Low | High | Researcher |  |
| Functionalities not clear | Some didn't know that they can choose their own workouts | Explain the app in an instructional video | Yes | Low | Low | Researcher |  |
|  | Favorite not clear, thought that with heart the exercise comes more often in training program | - Have favorites appear more often in the suggested program | Yes | Low | Low | Researcher |  |
|  | Possibilities filters not clear | Make the usefulness of the filters in the video clearer | Yes | Low | Low | Researcher |  |
|  |  | Make the usefulness of the filters in the overview clearer | No | Low | Low | Researcher |  |
| Not possible to indicate which workout she has already done | They want to see which workouts are new | Make your workout that hasn't been done visually visible | Yes | Low | Low | Engineer |  |
| Suggestion program | Workouts with grandchildren often don't work out | Workout with grandchildren not as a suggestion video but only in the overview | Yes | Low | Low | Researcher |  |
| A sense of community | There is no sense of belonging to a group | offering MIA in groups through, for example, the City of Hasselt | No | Low | Low | Researcher |  |
| Evaluation | Difficult for some, not for most | Clarify evaluation | Yes | Low | Low | Researcher |  |
| MIA feature: Continuous learning | | | | | | |  |
| clarity | not visible which articles have already been read | visualize when an article has been read | Yes | Low | High | Engineer |  |
| not read | Some haven't read anything | Highlight the usefulness of articles -- > intro movie? | Yes | Low | Low | Researcher |  |
| effect | Behavior not adjusted based on articles read |  | No | High | Low | Researcher |  |
| Content articles | Lots of ads on websites | Check websites and only use the good ones with no or fewer ads | No | High | Low | Researcher |  |
|  |  | More info in app instead of redirect website | No | High | Low | Researcher |  |
|  | too much info on websites |  | No | Low | Low | Researcher |  |
|  | too simple info |  | No | Low | Low |  |  |
|  | Not very newsworthy | Offering more innovative information | No | High | Low | Researcher |  |
|  | Long-form articles |  | No | Low | Low | Researcher |  |
| Themes | Also a theme around pensions | add | Yes | Low | Low | Researcher |  |
|  | Theme around dementia | add | Yes | Low | Low | Researcher |  |
|  | Theme on healthy ageing | add | Yes | Low | Low | Researcher |  |
|  | Keeping themes dynamic, so you can renew or change them in between |  | Yes | Low | Low | Researcher |  |
| errors | Lots of language errors | Checking content for language errors | Yes | Low | Low | Researcher |  |
| MIA feature: Diary | | | | | | |  |
| Input | Difficult to estimate time when walking |  | No | Low | Low | Researcher |  |
|  | Editing or deleting activities was not possible | Add feature to be able to delete or modify | Yes | Low | High | Engineer |  |
|  | Difficulty estimating intensity and effort | Would you like to explain this better? Intro or manual? | Yes | Low | High | Researcher |  |
| Activities | Gardening is lacking | add | Yes | Low | High | Researcher |  |
|  | Divide household chores based on effort |  | Yes | Low | High | Researcher |  |
|  | Exercise bike is missing |  | Yes | Low | High | Researcher |  |
| Ambiguous | Some didn't know that you could fill in activities yourself | make it clear in intro video | Yes | Low | High | Researcher |  |
|  | someone thought that Mia takes diary into account when putting together exercise program | Explain the usefulness of the diary more clearly. Intro video? | Yes | Low | High | Researcher |  |
| social | would be nice if you can share it with others | Ability to export and share a report of your feed | No | High | Low | Engineer |  |
|  | Bugfix diary |  | Yes | Low | High | Engineer |  |
| MIA feature: Progression monitor | | | | | | |  |
| Not viewed | Not everyone has watched it | Intro video? | No | Low | Low | Researcher |  |
|  | You have to go there consciously, 1x a week push message with weekly overview | Create a weekly overview and email or push it | No | High | Low | Engineer |  |
| Contents | Overview 30 days too short | Also offer the possibility to look back at the entire period | Yes | Low | High | Engineer |  |
|  | not clear what the 7-day period is | Display the dates somewhere | Yes | Low | High | Engineer |  |
|  |  | overview per week instead of per 7 days | No | Low | Low | Engineer |  |
|  | Smileys no added value |  | No | Low | Low | Researcher |  |
|  | Number of articles read not clear | better description, perhaps by category? | Yes | Low | High | Engineer |  |
| Effect | Doesn't have a stimulating effect (at least not for everyone) |  | No | High | Low | Researcher |  |
| MIA feature: Personal profile | | | | | | |  |
| Not yet personalized enough | Age setting not possible | Adapted workouts provided per age category (65-70, 70-80, 80+) | Yes | Low | High | Engineer |  |
|  | Max. exercise minutes (150- WHO) per week are not adjustable | Set default to WHO guidelines, but give participant the option to increase control minutes | Yes | Low | High | Engineer |  |
|  | In case of physical problems (e.g. neck pain or back pain), no adjustments possible | Giving the possibility to filter out certain WO that are difficult for certain physical problems | Yes | High | High | Engineer |  |
| Usefulness of profile not clear | Not everyone knew that your profile was decisive for the creation of the training program | Indicate more clearly in tutorial video that your profile ensures personalization of WO schedule | Yes | Low | High | Researcher |  |
| Reminder email | not always well received | Indicate during installation and in manual that mails may end up in SPAM box | No | Low | Low |  |  |
|  | Not necessary for everyone | People are already given the option in profile to indicate whether they want to receive a reminder email or not. Indicate more clearly in tutorial video? | Yes | Low | High | Researcher |  |
|  | Push notification on device would be better | Giving people the option in profile to choose between mail, push notification or a combination | No | High | Low | Engineer |  |
| Not daring to adapt | Some people indicated that they did not dare to change the profile | Clarify that adjusting the profile only leads to a more personalized training schedule | Yes | Low | High | Engineer |  |
| Term "Profile" not clear | Some people didn't know what profile meant | Changing the term to "personal settings" | No | Low | Low | Engineer |  |
| MIA feature: Ask a question | | | | | | |  |
| clarity | Helpdesk answer not visible via email | Send mail with a copy of the reply instead of only in the app | Yes | Low | High | Engineer |  |
| answer | No personal answers | Low | No | Low | Low | Researcher |  |
|  | No quick answer |  | No | Low | Low | Researcher |  |
| not used, not needed |  |  | No | Low | Low | Researcher |  |
| MIA feature: Lay Out | | | | | | |  |
| Clarity | Too small on mobile phone | Duplicate row 8 | No | High | Low | Engineer |  |
| Design | Old-fashioned/not flashy/boring/old-fashioned | Most thought layout was good, only in 1 focus group they were negative | No | High | Low | Engineer |  |
| MY feature: Homepage | | | | |  |  |  |
| Exercise Minutes/Goal | 150 minutes is too little, goal achieved quickly and that is not motivating | Make the minutes of exercise goal adjustable in profile | Yes | Low |  | Engineer |  |
|  | unclear what it is, which 7 days | Explain a sentence | No | Low |  | JB |  |
|  |  | Explain more clearly why this is important in profile | No | Low |  | Engineer |  |
|  | Usefulness of smileys unclear | Explain the usefulness of smileys better | Yes |  |  | Researcher |  |
| Tip of the day | changed too little | create a larger database with tips of the day + also provide practical tips about MIA | Yes |  |  | Researcher |  |
| Suggestion program | Now only visible for 3 days, better if that's a week | make a week visible instead of 3 days | No |  |  |  |  |
|  | Leave yesterday's exercise for if you haven't done it and want to catch up | Yesterday as well | No |  |  |  |  |
