# Supplementary material for: Exploring the Feasibility of a 5-Week mHealth Intervention to Enhance Physical Activity and an Active, Healthy Lifestyle in Community-Dwelling Older Adults: Mixed Methods Study
Source: JMIR Aging. 2025 Jan 27;8:e63348. doi: 10.2196/63348 (PMC11811674; doi:10.2196/63348)
Supplement: Multimedia Appendix 8 [file aging_v8i1e63348_app8.docx]

# Appendix 8: SWOT matrix of the MIA-app according to the NASSS components

|  | **Strengths** | **Weaknesses** | **Opportunities** | **Threats** |
| --- | --- | --- | --- | --- |
| Condition | | | | |
| n=30  17 female  70.3±4.8 years  70% married  76% with a higher degree  Digital literate (59.6±8.8 out of 74)  Wide range of PA level (IPAQ) | -Diverse gender representation  -Engagement of older adults is often overlooked in digital and fitness, filling a market gap.  -High digital literacy score suggesting that the target user base is capable of using digital tools. | -Complex needs of older adults  -Variability in PA levels | -Partnerships and collaborations  -Social features: leveraging the fact that many users really need social identification, the app could develop features that promote a buddy system to enhance motivation. | -Technological intimidation  -Health and mobility issues  -Competitive market |
| Technology | | | | |
| Web-Based mHealth app  SUS = 77.4±14.3  CSAT = 86,6%  NPS = 33,34 | -Engaging workout videos  -User interface and accessibility  -Personalization  -Features such as the chatbot and manual diary are highly valued by users  -Responsive support: Technical issues are resolved quickly, indicating strong customer support which can increase user satisfaction and trust | -Limited engagement with calendar activities  -Issues with editing the manual diary, short tracking periods in the progression monitor, and some navigational challenges point to areas needing usability enhancements.  -Insufficient communication on features: Users were unaware of certain functionalities indicating a need for clearer guidance | -Implementing a buddy system or enhancing the community calendar with more interactive features could increase user engagement and retention.  -Improvements in workout customization, expanded educational content | -Technological challenges |
| Value proposition | | | | |
|  | -Personalization  -Adaptability  Behavioral change techniques  -User centered design  -Community features | -Dependance on technology  -Complexity in customization | -Growing demand for mHealth solutions  -Partnerships with healthcare providers  -expansion to other markets  -Integration with healthcare data | - Rapid technological changes  User engagement challenges |
| Adopters | | | | |
| NPS = 33,34 with  50% Promotors  30% Passives  16.6 % Detractors | Good outcome on usability scales (NPS, CSAT, SUS)  50% promoters | -Dependance on user compliance | -Collaboration with insurance providers  -Support remote monitoring | -Detractors and also passives represent a churn risk  -Uncertain ROI |
| Organization | | | | |
|  | -Innovative and adaptive structure  -Strong support and training -Alignment with WHO goals | -Resource constraints  -Resistance to change  -Complexity in Integration | -Expansion and scaling  -Enhanced data utilization  -Grant and funding opportunities | -Technological disruptions  -Regulatory and compliance issues |
| Wider System | | | | |
|  | -Alignment with health promotion policies  -Demographic trends  -Support from stakeholders | -Dependence on external funding  -Regulatory challenges  -Market saturation | -Technological advancements  -Partnerships and collaborations  -Global Expansion to Low and Mid Income Countries | -Technological obsolescence  -Privacy and security concerns  -Policy and regulatory changes |
| Embedding and adaptation | | | | |
|  | -Continuous improvement possible thanks to easy design of MIA  -Adaptive Algorithms | -Challenges in long-term engagement  -Resource intensive | -Technological advances  -User retention through innovation | User fatigue |
