# Supplementary material for: Exploring the Feasibility of a 5-Week mHealth Intervention to Enhance Physical Activity and an Active, Healthy Lifestyle in Community-Dwelling Older Adults: Mixed Methods Study
Source: JMIR Aging. 2025 Jan 27;8:e63348. doi: 10.2196/63348 (PMC11811674; doi:10.2196/63348)
Supplement: Multimedia Appendix 9 [file aging_v8i1e63348_app9.docx]

# Appendix 9: Willingness to pay analysis

To assess the price acceptability of the MIA-app, a Van Westendorp price sensitivity analysis (1) was conducted. The objective was to determine a price range where most consumers consider the MIA-app cost reasonable, avoiding prices that are perceived as "too cheap" or "too expensive." The analysis revealed that the optimal price range for the MIA-app is between €4.50 and €7.00. This range reflects a balance between affordability and perceived value, where users are comfortable with the cost of the app.

Reference

1. Ceylana HH, Koseb B, Aydin M. Value based pricing: A research on service sector using Van Westendorp Price Sensitivity Scale. Procedia-Social and Behavioral Sciences. 2014;148:1-6.
